# Plasma p-tau212 identifies cognitively unimpaired individuals with emerging amyloid-β pathology

**DOI:** 10.1007/s00415-025-13572-5

**Published:** 2025-12-25

**Authors:** Przemysław R. Kac, Armand González-Escalante, Marta Milà-Alomà, Nicholas J. Ashton, Mahnaz Shekari, Paula Ortiz-Romero, Michael Turton, Peter Harrison, Henrik Zetterberg, Juan Domingo Gispert, Thomas K. Karikari, Marc Suárez-Calvet, Kaj Blennow

**Affiliations:** 1 Department of Psychiatry and Neurochemistry, Institute of Neuroscience and Physiology, The Sahlgrenska Academy at the University of Gothenburg, Mölndal, Sweden; 2 Barcelonaβeta Brain Research Center (BBRC), Pasqual Maragall Foundation, Barcelona, Spain; 3 Hospital del Mar Research Institute, Barcelona, Spain; 4 Universitat Pompeu Fabra, Barcelona, Spain; 5 Northern California Institute for Research and Education, San Francisco, CA USA; 6 UK NIHR Biomedical Research Centre for Mental Health and Biomedical Research Unit for Dementia at South London and Maudsley NHS Foundation, London, UK; 7 Centre for Age-Related Medicine, Stavanger University Hospital, Stavanger, Norway; 8 Banner Alzheimer’s Institute, University of Arizona, Phoenix, AZ USA; 9 Bioventix Plc, Farnham, UK; 10 Department of Neurodegenerative Disease, Dementia Research Centre, UCL Institute of Neurology, Queen Square, London, UK; 11 UK Dementia Research Institute, University College London, London, UK; 12 Hong Kong Center for Neurodegenerative Diseases, HKCeND, Hong Kong, China; 13 School of Medicine and Public Health, University of Wisconsin-Madison, Madison, USA; 14 Clinical Neurochemistry Laboratory, Sahlgrenska University Hospital, Mölndal, Sweden; 15 Centre for Brain Research, Indian Institute of Science, Bangalore, India; 16 Centro de Investigación Biomédica en Red Bioingeniería, Biomateriales y Nanomedicina, Madrid, Spain; 17 Department of Psychiatry, School of Medicine, University of Pittsburgh, Pittsburgh, USA; 18 Servei de Neurologia, Hospital del Mar, Barcelona, Spain

**Keywords:** Alzheimer’s disease, p-tau212, p-tau217, Amyloid PET, Aβ, Preclinical

## Abstract

Plasma phosphorylated-tau (p-tau) biomarkers show high performance as first-in-line tests in the evaluation of patients with cognitive symptoms and have also found utility in clinical trials for anti-amyloid drug therapies for Alzheimer’s disease (AD). Plasma p-tau212 is a novel blood biomarker that can discriminate cognitively unimpaired (CU) and subjective cognitive decline patients from mild cognitive impairment (MCI)-AD and AD dementia patients in clinical cohorts. Using Simoa technology, we evaluated plasma p-tau212 for its ability to detect emerging amyloid-beta (Aβ) pathology in CU individuals with brain amyloidosis from CSF Aβ42/40 ratio (*n* = 317) or Aβ PET scans (*n* = 277). We benchmarked plasma p-tau212 against p-tau181, p-tau217, and p-tau231 by comparing their accuracies and fold changes for detecting brain amyloidosis. Our results showed that all the plasma p-tau biomarkers are increased in the CU CSF Aβ + participants, with the highest median fold change observed for plasma p-tau212. Discrimination performance of the biomarkers to differentiate amyloid PET-positive from amyloid PET-negative participants differed between plasma p-tau biomarkers, being higher for plasma p-tau217 and p-tau212 compared to plasma p-tau181 and p-tau231. Furthermore, plasma p-tau212 was elevated in participants with low Aβ burden (CSF Aβ-positive, amyloid PET-negative), supporting its potential as a cost-effective, easily implemented biomarker for trial recruitment in early stages. Its further increase in Aβ PET-positive participants suggests additional utility for monitoring anti-amyloid therapies.

**Supplementary Information:**

The online version contains supplementary material available at 10.1007/s00415-025-13572-5.

## Introduction

Recent advances in amyloid-targeting antibodies for Alzheimer’s disease (AD)-modifying treatments have resulted in approval from the Food and Drug Administration (FDA) for aducanumab [1], donanemab [2], and lecanemab [3]. The European Medicines Agency (EMA) also approved donanemab and lecanemab [4, 5]. Appropriate use recommendations find it necessary to confirm amyloid-beta (Aβ) abnormalities either in cerebrospinal fluid (CSF) or in positron emission tomography (PET) scans [6]. However, the diagnosis of symptomatic AD is complex and not devoid of misdiagnosis [7]. Also, limited accessibility to the recommended techniques to confirm Aβ pathology is another challenge in primary and secondary care [8].

Aβ accumulation in the brain is present decades before the onset of dementia [9]. Those cognitively unimpaired (CU) but amyloid-positive people are classified as preclinical AD by the National Institute on Aging-Alzheimer’s Association (NIA-AA) criteria [10] or asymptomatic at-risk by the IWG [11]. Currently, there are trials that focus on using anti-amyloid drugs in the preclinical AD subpopulation [12].

Recent technological advancements allow us to detect Aβ abnormalities in CU people using blood biomarkers [13–16]. So far, the leading candidates for discriminating Aβ-positive from Aβ-negative individuals from blood tests are the phosphorylated tau (p-tau) species [17–20]. Their association with CSF and PET biomarkers was confirmed in multiple research articles [15, 19, 21, 22], making them ideal candidates for recruiting and enriching CU Aβ-positive participants in clinical trials. Indeed, their utility was already noticed in clinical trial design, where plasma p-tau217 was measured at the entry in CU participants in the donanemab clinical trial [23]. From the measurable p-tau species, plasma p-tau231 appears to be increased earlier in the AD *continuum* than others [15], yet recent developments in the field focus more on targeting the p-tau217 epitope, which provides better diagnostic accuracies, has higher median-fold changes, and is more accessible [24]. There are other available p-tau epitopes, including plasma p-tau181, which was the first p-tau measured in AD patients’ plasma [25], p-tau205 [24], an epitope with higher potential impact in later stages of the disease [26], and p-tau212 [19].

Similarly to other plasma p-tau species, p-tau212 is an autopsy-confirmed biomarker that can discriminate CU and subjective cognitive decline patients from mild cognitive impairment (MCI)-AD and AD dementia patients in clinical cohorts as well as asymptomatic Down syndrome (aDS) participants from prodromalDS (pDS) and dementiaDS (dDS) [19, 27]. Plasma p-tau212 has similar accuracy to the p-tau217 immunoassay in detecting CSF Aβ pathology in biomarker-positive participants [24]. However, the capacity of plasma p-tau212 to detect emerging Aβ pathology in CU individuals is unknown.

In this study, we measured plasma p-tau212 in CU individuals with underlying Aβ pathology, as indicated by abnormal CSF Aβ42/40 ratio and/or amyloid-PET. We have also benchmarked plasma p-tau212 against other commonly used plasma p-tau biomarkers by comparing the diagnostic accuracies and fold changes of plasma p-tau212 with other available biomarkers, i.e., p-tau181, p-tau217, and p-tau231.

## Methods

### Participants’ characteristics

The research was performed in the ALFA + cohort, which is a nested longitudinal study from the ALFA (for ALzheimer’s and FAmilies) study [28]. The ALFA study includes 2743 middle-aged, CU individuals (CDR = 0; MMSE ≥ 26; semantic fluency ≥ 12), with a high proportion of AD patients’ offspring and *APOE* ε4 carriership [29]. ALFA + participants are followed longitudinally every 3 years and are comprehensively characterized with clinical and neuropsychological evaluations, CSF and blood biomarkers measurements, and neuroimaging biomarkers, including magnetic resonance imaging (MRI) and amyloid PET. In this study, we included 317 participants who had available measurements for all plasma biomarkers (plasma p-tau181, p-tau212, p-tau217, and p-tau231). Among them, 277 participants had available [^18^F]flutemetamol (Aβ) PET scans, and 303 participants had available CSF measurements. Participants were classified as Aβ-positive (A +) if CSF Aβ42/40 < 0.071 and tau-positive (T +) if CSF Mid(M)-p-tau181 (measured on the Elecsys platform) pg/ml[30]. For Aβ PET comparisons, we used a well-established cut (A + : Aβ PET > 24 Centiloids). Similarly to previously published articles, we also classified participants according to their CSF/PET Aβ status reflecting low Aβ burden. The CL24 threshold was used for binary classification of Aβ PET status, while the CL30 threshold was applied specifically to stratify amyloid burden into low and overt categories. Therefore, the group with a low burden of Aβ pathology was defined as CSF Aβ-positive (i.e. Aβ42/40 < 0.071) but Aβ PET-negative (i.e., Aβ PET Centiloids < 30), and was compared with CSF/PET Aβ-negative (CSF Aβ42/40 ≥ 0.071 and Aβ PET Centiloids < 30) and CSF/PET Aβ-positive (CSF Aβ42/40 < 0.071 and Aβ PET Centiloids ≥ 30) groups [15, 31].

### CSF and plasma collection, processing, and storage

CSF collection, processing, and storage in the ALFA + study have been described previously [30]. Briefly, lumbar puncture was performed at the intervertebral space L3/L4, L4/L5, or L5/S1 using a standard needle, between 8 am and 12 pm, and participants had fasted for at least 8 h. CSF was collected into a 15‐ml sterile polypropylene tube (Sarstedt, Nümbrecht, Germany; cat. no. 62.554.502), aliquoted in volumes of 0.5 ml into sterile polypropylene tubes (0.5 ml Screw Cap Micro Tube Conical Bottom; Sarstedt, Nümbrecht, Germany; cat. no. 72.730.005), and immediately frozen at − 80 °C.

CSF Aβ40, Aβ42 were measured with the exploratory NTK robust immunoassays (Roche Diagnostic International Ltd) on a cobas e 411 analyzer or cobas e 601 module. CSF M-p-tau181 and M-t-tau (both corresponding to the mid-region (M) domain of tau protein) were measured using the electrochemiluminescence Elecsys Phospho-Tau (181P) CSF and total-tau CSF immunoassays, respectively, on a fully automated cobas e 601 module (Roche Diagnostics International Ltd) as described previously [16].

Procedures of blood sample collection and processing were described in a previous publication [16]. In brief, blood samples were obtained the same day of the lumbar puncture and, therefore, in fasting conditions. Whole blood was drawn with a 20 g or 21 g needle gauge into a 10‐ml ethylenediaminetetraacetic acid (EDTA) tubes (BD Hemogard 10 ml; K2EDTA; cat. no. 367525). Tubes were gently inverted 5–10 times and centrifuged at 2000 g for 10 min at 4 °C. The supernatant was aliquoted in volumes of 0.5 ml into sterile polypropylene tubes (Sarstedt Screw Cap Micro Tube; 0.5 ml; PP; ref. 72.730.105) and immediately frozen at − 80 °C. The samples were processed at room temperature. The time between collection and freezing of both CSF and plasma samples was < 30 min.

Measurements of plasma p-tau181, p-tau212, and p-tau231 were performed on Simoa HD-X (Quanterix) in singlicates, according to the previously published and validated methods at the Clinical Neurochemistry Laboratory, Sahlgrenska University Hospital, Mölndal (Sweden), by scientists blinded to participants’ clinical information [17–19]. Plasma p-tau217 measurements were provided by Eli Lilly and Company using the validated Meso Scale Discovery method [32].

### Amyloid‐β positron emission tomography acquisition and processing

Imaging acquisition and preprocessing protocols have been described previously [33]. Briefly, [^18^F]flutemetamol PET scans were conducted in a Siemens Biograph mCT, following a cranial CT scan for attenuation correction. Participants were injected with 185 MBq of [^18^F]flutemetamol, and four frames of 5 min each were acquired 90 min post‐injection. PET images were reconstructed in 4 frames × 5 min using the three-dimensional Ordered Subset Expectation Maximization algorithm by incorporating time of flight and point spread function modelling. Centiloid values were calculated from the mean values of the standard Centiloid target region (https://www.gaain.org/centiloid-project) and the whole cerebellum as the reference region using the transformation previously calibrated [33].

### Statistical analysis

We selected only the participants with all plasma biomarkers available and removed only the most extreme univariate outliers (+/− 3 IQR x Q3/Q1) for every plasma biomarker. Demographic characteristics between Aβ groups were compared using the Wilcoxon rank-sum test for continuous variables and the chi-squared (*χ*^2^) test for categorical ones. Plasma levels of p-tau181, p-tau212, p-tau217, and p-tau231 were compared across subgroups defined by CSF Aβ status, Aβ PET status, CSF Aβ/PET status, and CSF-defined AT classification. For comparisons based on CSF Aβ and Aβ PET status, Wilcoxon rank-sum tests were used. For the group comparisons based on CSF/PET Aβ status and AT classification, pairwise differences were assessed by the Dwass–Steel–Critchlow–Fligner test. We performed further tests, adjusting linear models by sex, age, body mass index (BMI), and renal function (estimated glomerular filtration rate [eGFR]) and the previously defined amyloid subgroups, and performed post hoc pairwise contrasts of the estimated marginal means of the plasma biomarkers extracted from the models. We performed multiple testing correction by controlling the false discovery rate with the Benjamini–Hochberg method. The linear models’ assumptions were checked for, and the outcome log_10_ transformed if appropriate. Receiver operating curves (ROC) curve analyses were performed to assess the discrimination performance of each plasma biomarker in detecting CSF-defined or PET-defined Aβ pathology. Comparisons between ROC curves were conducted using DeLong’s tests. All statistical analyses and plots were performed using R (version 4.4.1), with a two-sided significance level set at *α* = 0.05.

### Ethical clearance

The ALFA + study (ALFA-FPM-0311) was approved by the independent ethics committee ‘Parc de Salut Mar’, Barcelona, and registered at http://clinicaltrials.gov/ (identifier: NCT02485730). All participating subjects signed the study’s informed consent form, which had also been approved by the independent ethics committee ‘Parc de Salut Mar’, Barcelona.

## Results

### Plasma p-tau212 is increased in CSF Aβ-positive cognitively unimpaired individuals and has the largest fold change

**Fig. 1:**
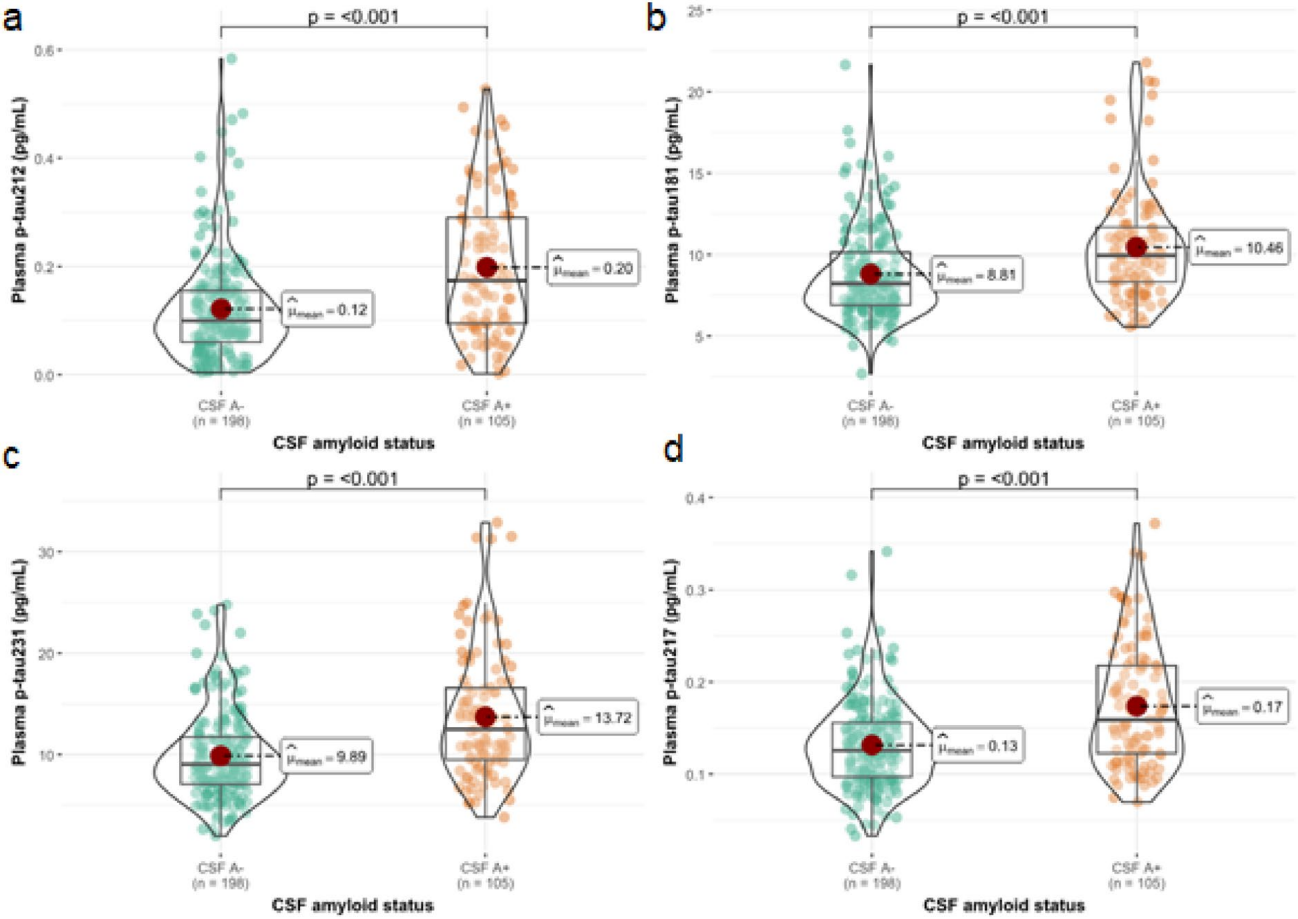
Plasma biomarkers in cognitively unimpaired individuals stratified by Aβ CSF status. The figure shows plasma biomarkers levels in participants classified according to their CSF Aβ status (Aβ-positive defined as CSF Aβ42/40 < 0.071). *N* = 198 cognitively unimpaired (CU) participants were classified as A-, and *n* = 105 CU participants were classified as A +. Plasma p-tau212 is shown in **a** and plasma p-tau181, p-tau231, and p-tau217 are shown in **b**, **c,** and **d,** respectively. Boxplots included in the violin plots show the median and interquartile range (IQR); the upper whisker is 75th percentile plus 1.5 times IQR and the lower whisker is 25th percentile minus 1.5 IQR. The red dot represents the mean of the group. Group differences were examined using the Wilcoxon rank-sum test

**Fig. 2:**
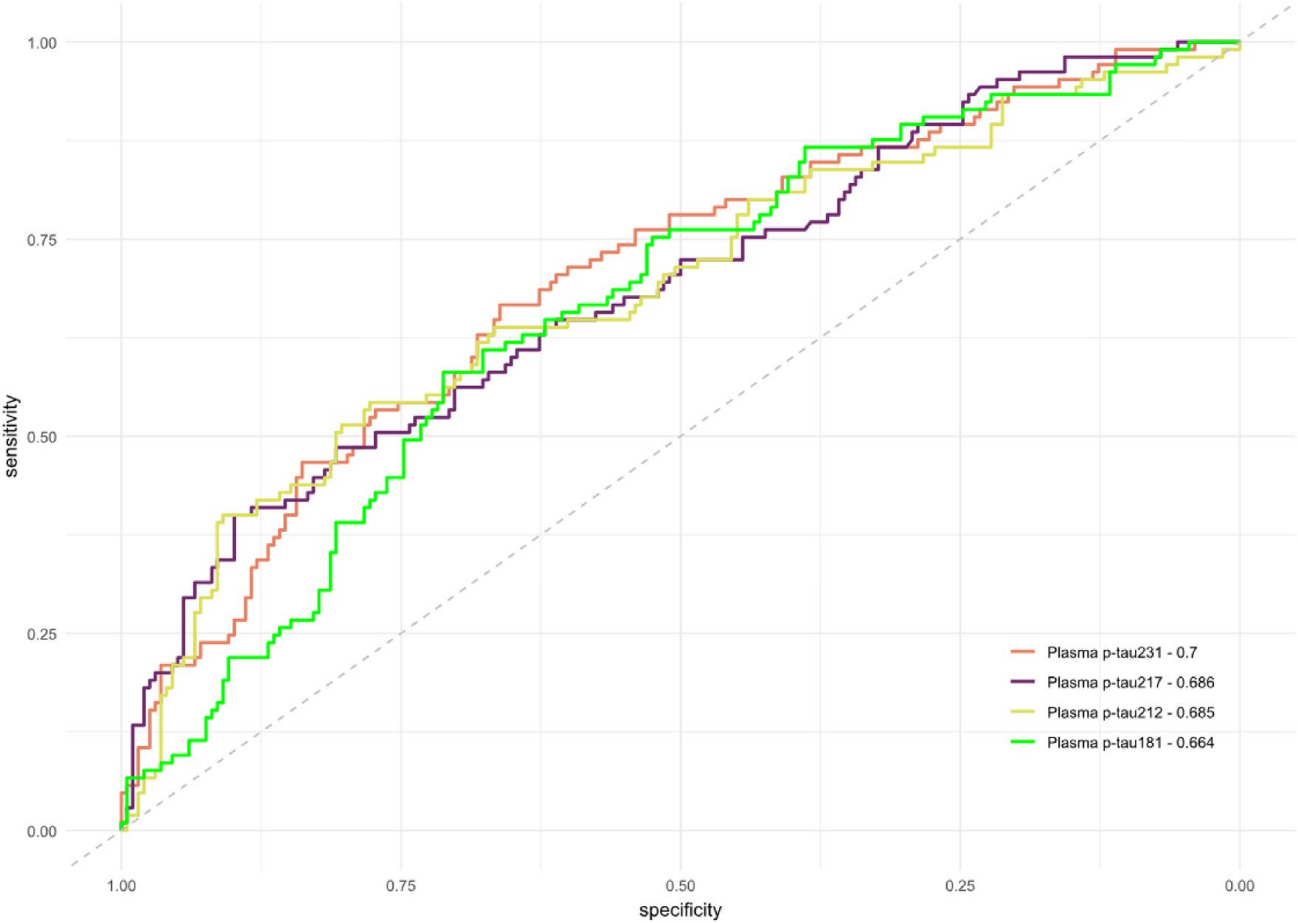
ROC analyses of plasma biomarkers for discriminating between CSF Aβ status. Receiver operating characteristic curves (ROC) to discriminate between cognitively unimpaired (CU) CSF A + and A- participants for the biomarkers presented in Fig. 1. p-tau181: AUC = 0.66 (95% CI 0.60–0.73); p-tau212: AUC = 0.68 (95% CI (0.62–0.75); p-tau217: AUC = 0.69 (95% CI 0.62–0.75) and p-tau231 AUC = 0.70 (95% CI 0.64–0.76); are on the graph

First, we classified participants by CSF Aβ status (Table 1). Levels of all the measured biomarkers were significantly increased in A + individuals when compared with A − participants. Plasma p-tau212 had the greatest median fold-change—1.70x (Fig. 1a; *p* < 0.001) compared with the other biomarkers (1.20 × for p-tau181; Fig. 1b; *p* < 0.001; 1.37 × for p-tau231; Fig. 1c; *p* < 0.001; 1.23 × for p-tau217; Fig. 1d; *p* < 0.001). The results remained unaltered in the models adjusted by covariates (Supplementary Fig. 1). The ability to discriminate between A + and A − participants was as follows: p-tau212 AUC = 0.68 (95% CI (0.62–0.75)), p-tau181 AUC = 0.66 (95% CI 0.60–0.73); p-tau231 AUC = 0.70 (95% CI 0.64–0.76); p-tau217 AUC = 0.69 (95% CI 0.62–0.75); CI 0.62–0.75); Fig. 2. DeLong’s test for AUC comparisons showed no statistically significant differences between these ROC curves. The effect size was moderate for all the biomarkers. Specifically, *r* = [−0.33 (−0.44—−0.20)] for p-tau181; *r* = [− 0.37 (− 0.48 to − 0.25)] for p-tau212; *r* = [− 0.40 (− 0.51 to − 0.28)] for p-tau231, and r = [− 0.37 (− 0.48 to − 0.25)] for p-tau217.

**Table 1:** Demographics of the study cohort stratified by CSF Aβ status

Characteristic	*N*	CSF A − *N* = 198	CSF A + *N* = 105	*p*-value
Age, Median (Q1, Q3)	303	60.3 (57.6, 63.9)	62.8 (58.4, 65.9)	0.017^1^
Sex, n (%)	303			0.3^2^
Men		77 (39%)	47 (45%)	
Women		121 (61%)	58 (55%)	
*APOE* ε4 carriership, n (%)	303			< 0.001^2^
Non-carrier		114 (58%)	24 (23%)	
Carrier		84 (42%)	81 (77%)	
MMSE, Median (Q1, Q3)	303	29.00 (29.00, 30.00)	29.00 (28.00, 30.00)	0.8^1^
BMI, Median (Q1, Q3)	303	26.3 (24.4, 29.8)	26.1 (24.0, 29.0)	0.6^1^
eGFR, Median (Q1, Q3)	301	93 (79, 109)	93 (75, 106)	0.4^1^
amyloid PET Centiloids, Median (Q1, Q3)	263	−4 (−9, 0)	9 (0, 24)	< 0.001^1^
CSF Aβ42 (pg/mL), Median (Q1, Q3)	303	1399 (1121, 1892)	821 (702, 1,048)	< 0.001^1^
CSF Aβ42/40, Median (Q1, Q3)	303	0.086 (0.081, 0.093)	0.053 (0.042, 0.061)	< 0.001^1^
CSF t-tau (pg/mL), Median (Q1, Q3)	303	173 (142, 214)	200 (163, 256)	< 0.001^1^
CSF p-tau181 Roche (pg/mL), Median (Q1, Q3)	303	13.7 (10.7, 17.7)	16.5 (13.1, 21.9)	< 0.001^1^
Plasma p-tau212 (pg/mL), Median (Q1, Q3)	303	0.10 (0.06, 0.15)	0.17 (0.09, 0.29)	< 0.001^1^
Plasma p-tau217 (pg/mL), Median (Q1, Q3)	303	0.13 (0.10, 0.16)	0.16 (0.12, 0.22)	< 0.001^*1*^
Plasma p-tau181 (pg/mL), Median (Q1, Q3)	303	8.22 (6.88, 10.17)	9.95 (8.31, 11.67)	< 0.001^1^
Plasma p-tau231 (pg/mL), Median (Q1, Q3)	303	9.1 (7.1, 11.7)	12.5 (9.5, 16.6)	< 0.001^1^

Numerical variables are presented as median and interquartile range (IQR: Q1–Q3), while categorical variables are shown as counts and percentages (%). Differences in numerical variables between CSF-defined Aβ groups were assessed using the Wilcoxon rank-sum test. For categorical variables, group differences were evaluated using Pearson’s chi-squared (*χ*^2^) test. Participants are classified based on their CSF Aβ status (Aβ-positive defined as CSF Aβ42/40 < 0.071.)

*Aβ* amyloid beta, *APOE*4 Apolipoprotein E4, *BMI* Body Mass Index, *CSF* Cerebrospinal Fluid, *eGFR* estimated Glomerular Filtration Rate, *MMSE* Mini–Mental State Examination, *p-tauX* tau phosphorylated at amino acid X, *PET* Positron Emission Tomography, *t-tau* total-tau

^1^Wilcoxon rank sum test

^2^Pearson’s Chi-squared test

### Plasma p-tau212 is elevated in Aβ PET-positive cognitively unimpaired individuals and has the largest fold change

**Fig. 3:**
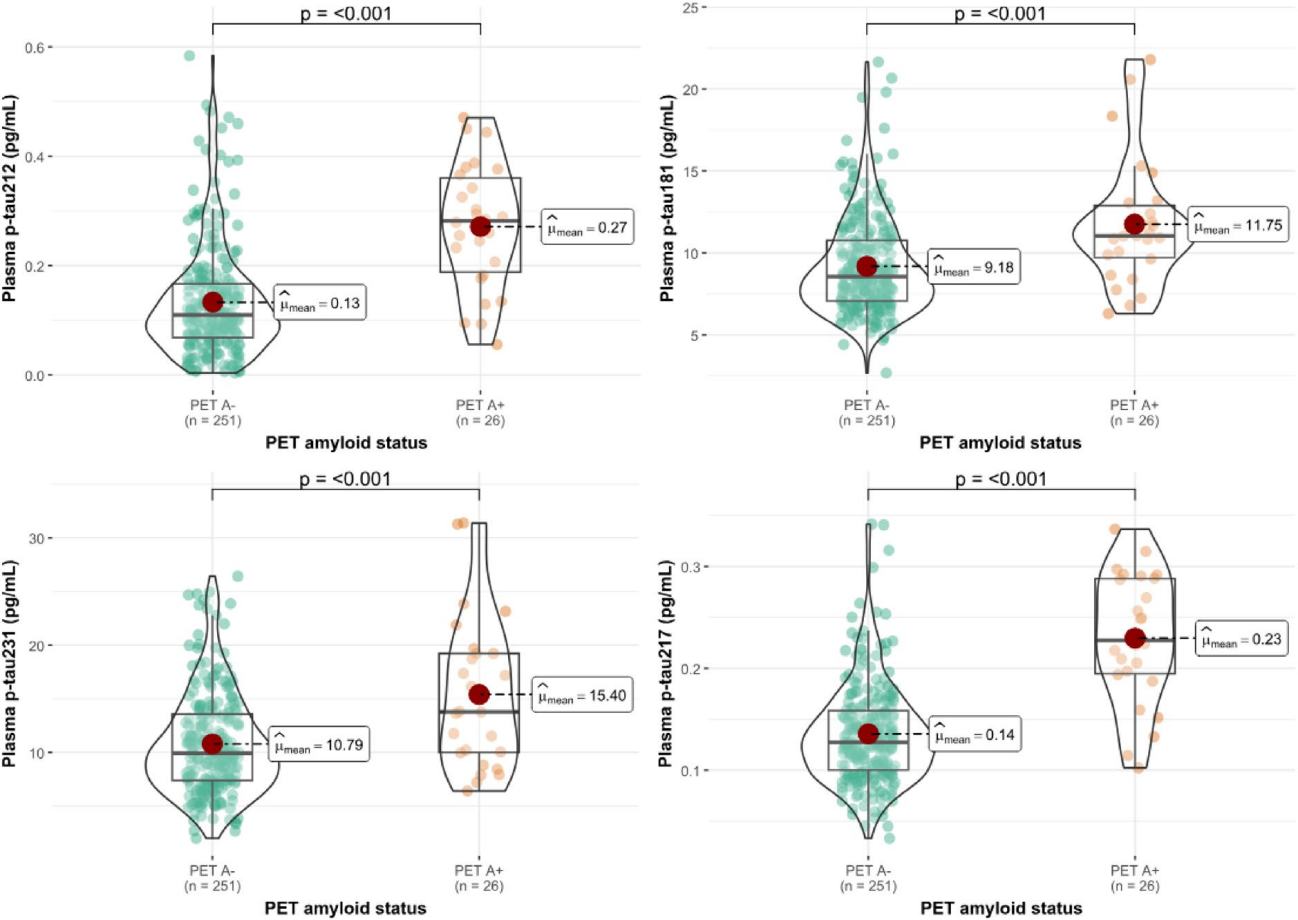
Plasma biomarkers in cognitively unimpaired individuals stratified by Aβ PET status. The figure shows plasma levels of the plasma biomarkers in participants classified according to their amyloid-PET status (A + : Aβ PET > 24 Centiloids). *N* = 251 cognitively unimpaired (CU) participants were classified as A-, and *n* = 26 CU participants were classified as A +. Plasma p-tau212 is shown in **a** and plasma p-tau181, p-tau231, and p-tau217 are shown in **b**, **c,** and **d,** respectively. Boxplots included in the violin plots are shown as a median and interquartile rangsse (IQR), the upper whisker is 75th percentile plus 1.5 times IQR and the lower whisker is 25th percentile minus 1.5 IQR. The red dot represents the mean of the group. Group differences were examined using the Wilcoxon rank-sum test

**Fig. 4:**
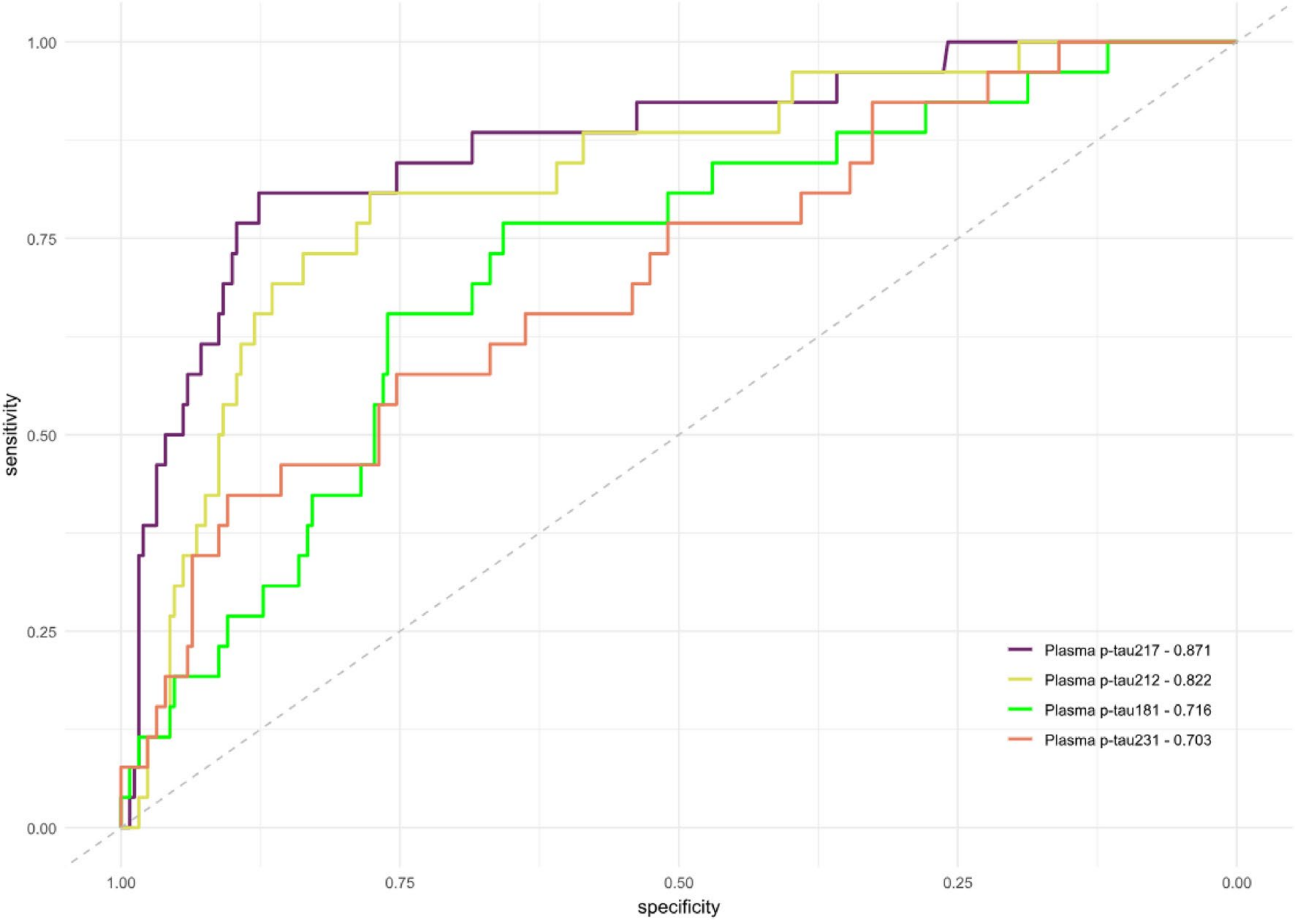
ROC analyses of plasma biomarkers for discriminating between PET Aβ status. Receiver operating characteristic curves (ROC) to discriminate between cognitively unimpaired (CU) CSF A + and A- participants for the biomarkers presented in Fig. 1. p-tau181: AUC = 0.72 (95% CI 0.61–0.82); p-tau212: AUC = 0.82 (95% CI 0.74–0.91); and p-tau217: AUC = 0.87 (95% CI 0.79–0.95); and p-tau231: AUC = 0.70 (95% CI 0.59–0.81) are on the graph

Next, we classified participants by their Aβ PET status (A + > 24 Centiloids; Supplementary Table 1). All the biomarkers were significantly higher in the PET A + participants compared with the Aβ PET A- participants. The results remained unaltered in the models adjusted by covariates (Supplementary Fig. 2). Similarly to the CSF results, plasma p-tau212 had the highest median fold-change (2.55x; *p* < 0.001; Fig. 3a), followed by plasma p-tau217 (1.77x; *p* < *0.001;* Fig. 3d), p-tau231 (1.4x; *p* < 0.001; Fig. 3c), and p-tau181 (1.29x; *p* < *0.0001;* Fig. 3b). We compared the AUC of the plasma biomarkers, and we observed significantly higher performance for plasma p-tau212 and p-tau217 when compared with plasma p-tau181 and p-tau231. Plasma p-tau217 was significantly better in discriminating Aβ PET A + individuals than p-tau181 (AUC = 0.87 [95% CI 0.79–0.95] vs AUC = 0.72 [95% CI 0.61–0.82]; *p* = 0.017) and from p-tau231 (AUC = 0.87 [95% CI 0.79–0.95] vs AUC = 0.70 [95% CI 0.59–0.81]; *p* = 0.001). There was no difference between p-tau217 and p-tau212 (AUC = 0.82 [95% CI 0.74–0.91]; *p* = 0.22). Still, plasma p-tau212 had significantly better diagnostic accuracy than plasma p-tau181 (*p* = 0.025) and p-tau231 (*p* = 0.0042) (Fig. 4). The superiority of p-tau212 and p-tau217 over p-tau181 and p-tau231 was also reflected in effect size, as prior had large effect size *r* = [− 0.64 (− 0.76 to − 0.49)] for p-tau212 and *r* = [− 0.74 (− 0.83 to − 0.62)] for p-tau217 compared to moderate *r* = [− 0.43 (− 0.60 to − 0.23)] for p-tau181 and *r* = [− 0.41 (− 0.58 to − 0.19)] for p-tau231.

### Plasma p-tau212 is increased in A + T- and A + T + CU participants

**Fig. 5:**
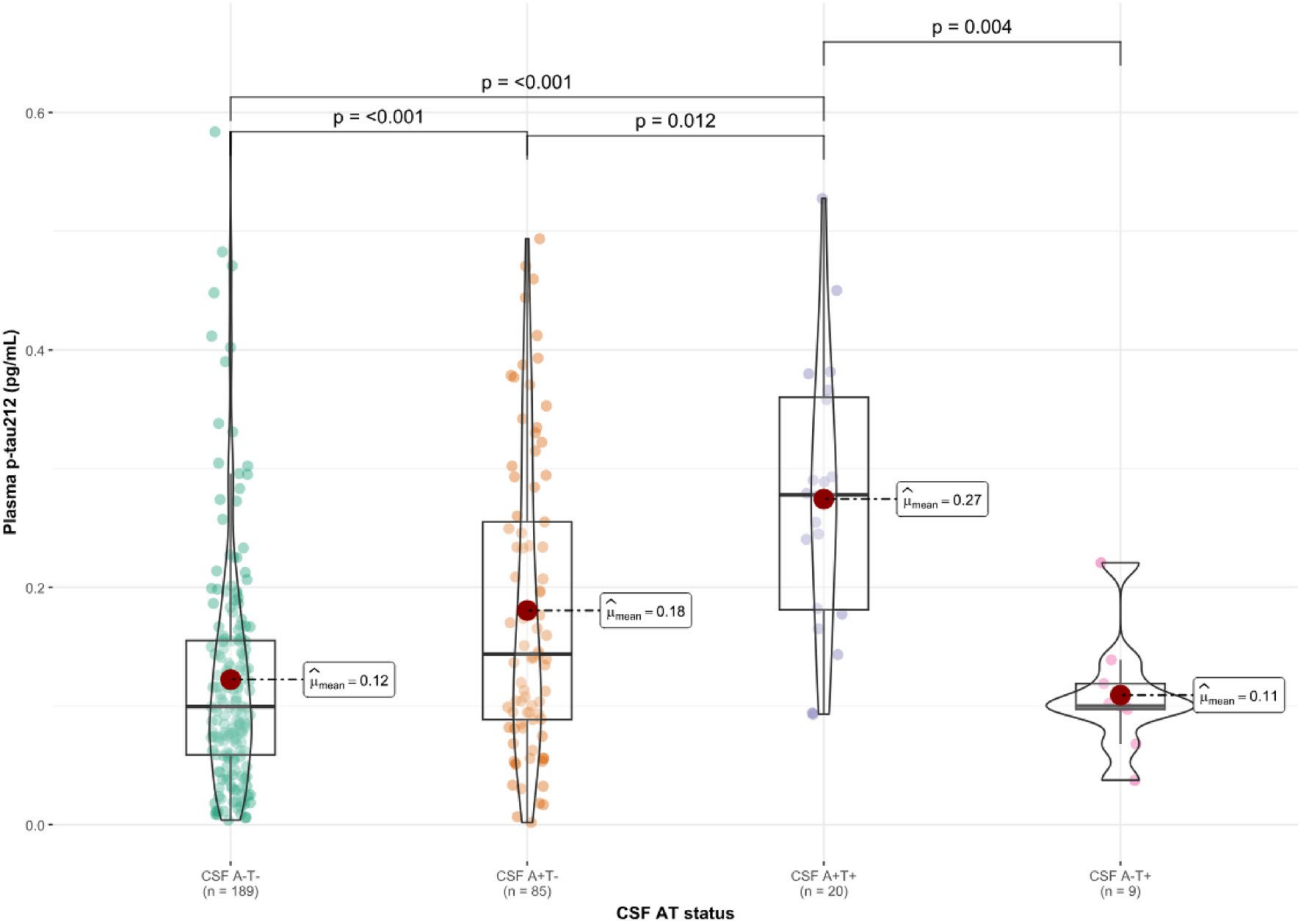
Plasma p-tau212 in cognitively unimpaired participants across CSF AT status. The figure shows plasma p-tau212 levels in participants classified according to their CSF AT status (A + Aβ42/40 < 0.071; T + M-p-tau181 > 24 pg/ml). *N* = 189 cognitively unimpaired (CU) participants were classified as A-T-; *n* = 85 CU participants were classified as A + T-; *n* = 20 CU participants were classified as A + T +; *n* = 9 CU participants were classified as A-T +. Boxplots included in the violin plots are shown as a median and interquartile range (IQR), the upper whisker is 75th percentile plus 1.5 times IQR and lower whisker is 25th percentile minus 1.5 IQR. The red dot represents the mean of the group. Pairwise differences were examined using the Dwass-Steel-Critchlow-Fligner test

Next, we classified our groups according to CSF AT status (A + : Aβ42/40 < 0.071 T + : M-p-tau181 > 24 pg/mL; Supplementary Table 2). All plasma biomarkers were increased in the A + T- group compared to the A-T- group (*p* < 0.001 for p-tau212 (Fig. 5), p-tau217 (Supplementary Fig. 5), and p-tau231 (Supplementary Fig. 4) and *p* = 0.002 for p-tau181 (Supplementary Fig. 3). Compared to the A-T- group, all plasma p-tau biomarkers were increased in the A + T + participants (*p* < 0.001, Fig. 5; and Supplementary Figs. 3–5). When A-T- participants were compared to A-T + participants, we observed no significant differences in the plasma biomarker levels (*p* = 0.843–0.999; Fig. 5; Supplementary Figs. 3–5). There were no significant differences when A-T + participants were compared to A + T- (*p* = 0.184–0.971), but we observed increased levels of p-tau212 (*p* = 0.002; Fig. 5) and p-tau217 (*p* = 0.047; Supplementary Fig. 5) in the A + T + group compared to the A-T + group. Plasma p-tau181 and p-tau231 were not statistically different for those groups’ comparison (Supplementary Figs. 3, 4). Additionally, we observed increased levels of plasma p-tau212 (*p* = 0.012; Fig. 5) and p-tau231 (*p* = 0.002; Supplementary Fig. 4) in A + T + compared to A + T- participants. In the models adjusted by covariates (Supplementary Figs. 6–9), we observed changes for p-tau217, where levels in A + T- became significantly lower than in the A + T + group (*p* = 0.038), and higher in A + T- when compared with A-T + group (*p* = 0.045) (Supplementary Fig. 8). Additionally, p-tau231 levels in A + T + group became significantly higher when compared with A-T + group (*p* = 0.009) (Supplementary Fig. 9).

### Plasma p-tau212 is increased in participants with low Aβ burden

**Fig. 6:**
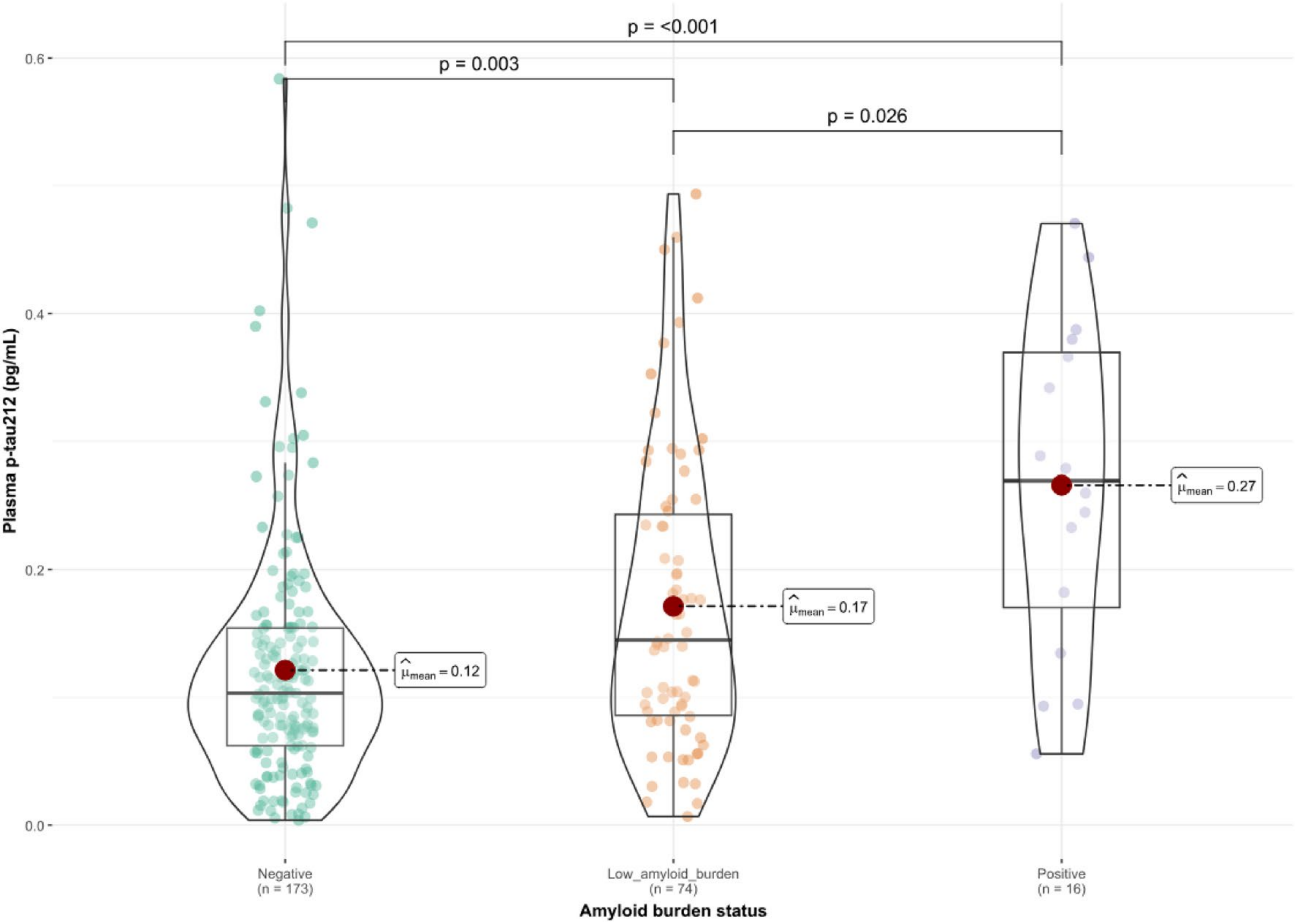
Plasma p-tau212 in cognitively unimpaired individuals accross Aβ CSF/PET status. The figure shows plasma p-tau212 levels in participants classified according to their Aβ burden status (CSF A +—Aβ42/40 < 0.071, PET A + > 30 Centiloids). *N* = 173 cognitively unimpaired (CU) participants were classified as negative; *n* = 74 CU participants were classified as low amyloid burden; *n* = 16 CU participants were classified as positive. Boxplots included in the violin plots are shown as a median and interquartile range (IQR), the upper whisker is 75th percentile plus 1.5 times IQR and the lower whisker is 25th percentile minus 1.5 IQR. The red dot represents the mean of the group. Pairwise differences were examined using the Dwass-Steel-Critchlow-Fligner test

At last, we classified people by their Aβ burden (CSF A +—Aβ42/40 < 0.071, PET A- < 30 Centiloids; Supplementary Table 3). All the plasma p-tau were significantly increased in the low Aβ burden group compared to the CSF/PET Aβ-negative group (*p* < 0.001–0.011; Fig. 6; Supplementary Figs. 10–12). We observed a further increase of p-tau212 (*p* = 0.026; Fig. 6) and p-tau217 (*p* < 0.001; Supplementary Fig. 12) when we compared low Aβ burden participants with CSF/PET Aβ-positive participants. All the biomarkers were increased in the CSF/PET Aβ-positive group when compared with the negative group (*p* < 0.001–0.004; Fig. 6; Supplementary Figs. 10–12). In the models adjusted by covariates, we only observed changes for p-tau231, where biomarker levels had significantly higher values in CSF/PET Aβ-positive participants when compared with low Aβ burden participants (*p* = 0.019) (Supplementary Figs. 13–16). We observed a 1.4 × median-fold increase of p-tau212 in the low Aβ burden group when compared to Aβ-negative groups. For plasma p-tau181, p-tau231, and p-tau217, we observed 1.1x, 1.4x, and 1.2 × median-fold change, respectively. Median levels in the CSF/PET-positive group were increased for plasma p-tau212, p-tau181, p-tau231, and p-tau217 2.7 ×, 1.3 ×, 1.9 ×, and 2.2 ×, respectively, when compared with CSF/PET Aβ-negative participants. ROC curve analyses to discriminate A- participants from the low Aβ burden group showed the best performance for p-tau231, followed by p-tau212, p-tau181, and p-tau217 (Supplementary Fig. 17).

## Discussion

In this study, we demonstrate that plasma p-tau212 is a promising biomarker for detecting Aβ pathology in CU individuals, potential candidates for AD prevention trials, and shows early increases along the disease continuum. Specifically, we found that plasma p-tau212 levels were elevated in CU individuals with Aβ pathology, as defined by both CSF and PET measures. Among the plasma biomarkers evaluated (p-tau181, p-tau217, and p-tau231), p-tau212 showed the largest fold changes. Moreover, plasma p-tau212 was already increased at very early stages of the Alzheimer’s continuum, including in individuals who were A + T − or had a low amyloid burden. However, the discriminative performance of plasma p-tau212 for identifying CSF Aβ positivity was modest (AUC = 0.685), though it improved when predicting PET-defined Aβ status (AUC = 0.822). The performance of p-tau212 was comparable to that of plasma p-tau217 (AUC = 0.871), with no statistically significant difference between them.

The rapid development of blood-based biomarkers for AD has had a positive impact due to the lack of accessibility and perceived invasiveness of the previous techniques, expanding possibilities of AD diagnosis, treatment, disease monitoring, clinical trials recruitment, or early intervention in the disease. Data acquired from clinical trials for AD disease-modifying therapies has provided consistent evidence for the increase of plasma p-tau181 and p-tau217 species in the placebo groups and a decrease of those in the drug treatment groups, along with a positive correlation with a reduction of amyloid in PET scans by the end of the trials [34]. The consideration that plasma biomarkers might be a replacement for amyloid PET scans or CSF biomarkers for AD diagnosis in the future is a possibility [14]. However, there are still some knowledge gaps to address. We are still uncertain about how sex, ethnicity, socioeconomic status, and comorbidities influence p-tau levels [35]. In this regard, we included available confounders such as sex, age, body mass index, and renal function (measured by eGFR) in our analyses. More research coming from real-life settings is lacking [34]; the use of the biomarkers in primary and/or secondary care is being debated, and the determination of standard procedures and cutoff points need to be conclusive [7, 8, 14, 35]. Plasma p-tau212 shows strong associations with amyloid pathology, but current evidence does not support consistent elevations in non-AD tauopathies. Similar to other soluble phosphorylated tau biomarkers, p-tau212 likely reflects tau changes occurring downstream of amyloid deposition, consistent with its classification as a T1 biomarker in the 2024 NIA-AA framework. Therefore, more research from a broad spectrum of blood-based biomarkers tests in the AD field is necessary to acquire sufficient information.

This research provides additional data about the use of biomarkers in cognitively unimpaired individuals at risk and, hence, candidates for prevention studies. Plasma p-tau212, a relatively new autopsy-validated biomarker, has already been shown to have excellent diagnostic accuracy in memory clinic cohorts [19], Down Syndrome and sporadic AD cohorts [36], including in autopsy-confirmed cases [19]. Additionally, a direct comparison of this biomarker in the GBSC Round Robin study showed that median fold changes of this biomarker in the biomarker-positive cohort are similar to the p-tau217 immunoassays in plasma and CSF [24].

Our findings support previous similarities between plasma p-tau212 and p-tau217, and their superior performance to discriminate amyloid-PET-negative participants from amyloid-PET-positive; however, all the biomarkers performed similarly when CSF positivity was included. Since most of the clinical trials for AD do not target amyloid or tau [37], p-tau212 might find use in specific clinical settings or clinical trials where measurements of other p-tau species would perform worse or would need to be excluded. Intriguingly, p-tau212 has specific mechanisms underlying its phosphorylation and dephosphorylation [19, 38, 39]. There are several drugs targeting protein kinases for preclinical AD stages [40]. From those, the dual-specificity tyrosine phosphorylation-regulated kinase 1 A (DYRK1A) appears to be the most specific kinase related to p-tau212 [41]. DYRK1A is mechanistically involved in Aβ plaques and neurofibrillary tangles formation, and influences cognition and memory dysfunctions in AD [42–44]. P-tau212 was proven to respond to lower doses of DYRK1A than p-tau181, p-tau217, and p-tau231; notably also for p-tau205 or p-tau262 [41]. P-tau212, through its early response to the DYRK1A, has been used to test the efficacy of kinase inhibitors in cell and animal models [45, 46]. To this end, p-tau212 was already observed to decrease after treatment with an AD drug candidate [47] and therefore has gained attention as a potential target engagement biomarker in clinical trials [48]. Certain p-tau217 immunoassays also cross-react with p-tau212, showing great discriminative accuracy [49].

Therefore, information about p-tau212 levels in preclinical studies might also help with the exclusion/inclusion criteria for clinical trials, which will include p-tau212 as a target engagement biomarker for AD therapies that indirectly target amyloid or tau pathology in AD, and an increase of this epitope might reflect a multifactorial imbalance in homeostasis. The fact that in several cases an increase of p-tau212 in the memory clinic cohort was previously observed without an increase of p-tau217 [19] might be supporting this hypothesis. Nevertheless, plasma p-tau212 should be interpreted within its clinical context. Although it reliably detects AD-related biological changes in cognitively unimpaired individuals, biomarker positivity alone does not imply inevitable disease progression. Plasma biomarkers currently indicate the presence of pathology, not its clinical trajectory. Population-based screening is not yet supported but remains a promising target for future research.

Together these results confirm the utility of plasma p-tau212 in differentiating CU amyloid-positive from CU amyloid-negative participants, providing valuable information for the practical use of that biomarker in clinical trial settings and in clinics.

This study is not devoid of limitations. Plasma p-tau212 needs to be investigated in diverse populations. The ALFA + cohort represents a selected population with elevated risk for AD, due to family history of sporadic AD. Therefore, our findings reflect biomarker behavior in preclinical AD (CU A + individuals) but do not allow conclusions about biomarker performance for detecting preclinical AD in the general population or for use in population-based screening. The low accessibility of the immunoassay is another limitation of this biomarker; however, considering its recently validated utility and use in multiple contexts, we might expect the appearance of other immunoassays targeting this epitope. Another limitation is the absence of tau-PET imaging to confirm brain tau pathology and assess the association of plasma p-tau212 with insoluble tau deposition; however, the relative performance of the biomarkers will be confounded by the relatively early stage of the cohort. Finally, the performance of the p-tau217 to discriminate from CSF or amyloid-PET-positive participants might be underestimated in the present study as it did not reach as high performance as in previously published articles [50–52]. This, however, might be the result of the strict settings used in this study.

## Conclusions

The increase of plasma p-tau212 before established Aβ PET positivity makes it a cost-effective and simple-to-implement biomarker for clinical trial recruitment purposes with potential multi-utility. Further increase of p-tau212 in Aβ PET-positive participants suggests that this biomarker has utility for clinical monitoring of anti-amyloid therapies as well.

## Supplementary Information

Below is the link to the electronic supplementary material.

Supplementary file1 (DOCX 3741 KB)

## Data Availability

Blinded Anonymized data is available on reasonable request from the corresponding author. Request will be reviewed by the investigators and respective institutions to verify if data transfer is in the agreement with EU legislation on the general data protection or is subject to any intellectual property or confidentiality obligations.

## References

[1] O. of the Commissioner (2024) FDA Grants Accelerated approval for Alzheimer’s Drug. FDA. [Online]. Available: https://www.fda.gov/news-events/press-announcements/fda-grants-accelerated-approval-alzheimers-drug. Accessed 18 Dec 2024

[2] C. for D. E. and Research (2024) FDA approves treatment for adults with Alzheimer’s disease. FDA. [Online]. Available: https://www.fda.gov/drugs/news-events-human-drugs/fda-approves-treatment-adults-alzheimers-disease. Accessed 18 Dec 2024

[3] O. of the Commissioner (2023) FDA grants accelerated approval for Alzheimer’s disease treatment. FDA. [Online]. Available: https://www.fda.gov/news-events/press-announcements/fda-grants-accelerated-approval-alzheimers-disease-treatment. Accessed 8 May 2023

[4] Health and Food Safety - Commission authorises medicine for treatment of early Alzheimer’s disease. [Online]. Available: https://ec.europa.eu/newsroom/sante/items/879055/en. Accessed 30 Apr 2025

[5] Kisunla | European Medicines Agency (EMA). [Online]. Available: https://www.ema.europa.eu/en/medicines/human/EPAR/kisunla. Accessed 17 Sep 2025

[6] Cummings J et al (2023) Lecanemab: appropriate use recommendations. J Prev Alzheimers Dis 10(3):362–377. 10.14283/jpad.2023.30 37357276 10.14283/jpad.2023.30 PMC10313141

[7] Schindler SE et al (2024) Acceptable performance of blood biomarker tests of amyloid pathology — recommendations from the Global CEO Initiative on Alzheimer’s Disease. Nat Rev Neurol 20(7):426–439. 10.1038/s41582-024-00977-5 38866966 10.1038/s41582-024-00977-5

[8] Palmqvist S et al (2024) Blood biomarkers to detect Alzheimer disease in primary care and secondary care. JAMA 332(15):1245–1257. 10.1001/jama.2024.13855 39068545 10.1001/jama.2024.13855 PMC11284636

[9] Jansen WJ et al (2015) Prevalence of cerebral amyloid pathology in persons without dementia: a meta-analysis. JAMA 313(19):1924–1938. 10.1001/jama.2015.4668 25988462 10.1001/jama.2015.4668 PMC4486209

[10] Jack CR et al (2018) NIA-AA research framework: toward a biological definition of Alzheimer’s disease. Alzheimers Dement 14(4):535–562. 10.1016/j.jalz.2018.02.018 29653606 10.1016/j.jalz.2018.02.018 PMC5958625

[11] Dubois B et al (2024) Alzheimer disease as a clinical-biological construct—an international working group recommendation. JAMA Neurol 81(12):1304–1311. 10.1001/jamaneurol.2024.3770 39483064 10.1001/jamaneurol.2024.3770 PMC12010406

[12] Rafii MS et al (2023) The AHEAD 3–45 study: design of a prevention trial for Alzheimer’s disease. Alzheimers Dement 19(4):1227–1233. 10.1002/alz.12748 35971310 10.1002/alz.12748 PMC9929028

[13] Therriault J et al (2024) Diagnosis of Alzheimer’s disease using plasma biomarkers adjusted to clinical probability. Nat Aging 4(11):1529–1537. 10.1038/s43587-024-00731-y 39533113 10.1038/s43587-024-00731-y PMC11564087

[14] Hansson O, Blennow K, Zetterberg H, Dage J (2023) Blood biomarkers for Alzheimer’s disease in clinical practice and trials. Nat Aging 3(5):506–519. 10.1038/s43587-023-00403-3 37202517 10.1038/s43587-023-00403-3 PMC10979350

[15] Milà-Alomà M et al (2022) Plasma p-tau231 and p-tau217 as state markers of amyloid-β pathology in preclinical Alzheimer’s disease. Nat Med 28(9):1797–1801. 10.1038/s41591-022-01925-w 35953717 10.1038/s41591-022-01925-w PMC9499867

[16] Suárez‐Calvet M et al (2020) Novel tau biomarkers phosphorylated at T181, T217 or T231 rise in the initial stages of the preclinical Alzheimer’s continuum when only subtle changes in Aβ pathology are detected. EMBO Mol Med. 10.15252/emmm.202012921 33169916 10.15252/emmm.202012921 PMC7721364

[17] Karikari TK et al (2020) Blood phosphorylated tau 181 as a biomarker for Alzheimer’s disease: a diagnostic performance and prediction modelling study using data from four prospective cohorts. Lancet Neurol 19(5):422–433. 10.1016/S1474-4422(20)30071-5 32333900 10.1016/S1474-4422(20)30071-5

[18] Ashton NJ et al (2021) Plasma p-tau231: a new biomarker for incipient Alzheimer’s disease pathology. Acta Neuropathol 141(5):709–724. 10.1007/s00401-021-02275-6 33585983 10.1007/s00401-021-02275-6 PMC8043944

[19] Kac PR et al (2024) Plasma p-tau212 antemortem diagnostic performance and prediction of autopsy verification of Alzheimer’s disease neuropathology. Nat Commun 15(1):2615. 10.1038/s41467-024-46876-7 38521766 10.1038/s41467-024-46876-7 PMC10960791

[20] Palmqvist S et al (2020) Discriminative accuracy of plasma phospho-tau217 for Alzheimer disease vs other neurodegenerative disorders. JAMA 324(8):772–781. 10.1001/jama.2020.12134 32722745 10.1001/jama.2020.12134 PMC7388060

[21] Smirnov DS et al (2022) Plasma biomarkers for Alzheimer’s disease in relation to neuropathology and cognitive change. Acta Neuropathol 143(4):487–503. 10.1007/s00401-022-02408-5 35195758 10.1007/s00401-022-02408-5 PMC8960664

[22] Lantero Rodriguez J et al (2020) Plasma p-tau181 accurately predicts Alzheimer’s disease pathology at least 8 years prior to post-mortem and improves the clinical characterisation of cognitive decline. Acta Neuropathol 140(3):267–278. 10.1007/s00401-020-02195-x 32720099 10.1007/s00401-020-02195-x PMC7423866

[23] Eli Lilly, Company (2024) A Study of Donanemab Versus Placebo in Participants at Risk for Cognitive and Functional Decline of Alzheimer's Disease’, clinicaltrials.gov, Clinical trial registration NCT05026866. [Online]. Available: https://clinicaltrials.gov/study/NCT05026866. Accessed 02 Jan 2025

[24] Ashton NJ et al (2024) The Alzheimer’s Association Global Biomarker Standardization Consortium (GBSC) plasma phospho-tau Round Robin study. medRxiv. 10.1101/2024.08.22.24312244 10.1002/alz.14508 PMC11851162 39907496

[25] Tatebe H et al (2017) Quantification of plasma phosphorylated tau to use as a biomarker for brain Alzheimer pathology: pilot case-control studies including patients with Alzheimer’s disease and down syndrome. Mol Neurodegener 12:63. 10.1186/s13024-017-0206-8 28866979 10.1186/s13024-017-0206-8 PMC5582385

[26] Jack CR Jr et al (2024) Revised criteria for diagnosis and staging of Alzheimer’s disease: Alzheimer’s Association workgroup. Alzheimers Dement 20(8):5143–5169. 10.1002/alz.13859 38934362 10.1002/alz.13859 PMC11350039

[27] Kac PR et al (2025) Plasma p-tau212 as a biomarker of sporadic and Down syndrome Alzheimer’s disease. Alzheimers Dement 21(4):e70172. 10.1002/alz.70172 40275833 10.1002/alz.70172 PMC12022499

[28] Molinuevo JL et al (2016) The ALFA project: a research platform to identify early pathophysiological features of Alzheimer’s disease. Alzheimer’s Dement: Trans Res Clin Interv 2(2):82–92. 10.1016/j.trci.2016.02.003 10.1016/j.trci.2016.02.003 PMC5644283 29067295

[29] Vilor‐Tejedor N et al (2023) Genetic characterization of the ALFA study: uncovering genetic profiles in the Alzheimer’s continuum. Alzheimers Dement 20(3):1703–1715. 10.1002/alz.13537 38088508 10.1002/alz.13537 PMC10984507

[30] Milà‐Alomà M et al (2020) Amyloid beta, tau, synaptic, neurodegeneration, and glial biomarkers in the preclinical stage of the Alzheimer’s continuum. Alzheimers Dement 16(10):1358–1371. 10.1002/alz.12131 32573951 10.1002/alz.12131 PMC7586814

[31] Milà-Alomà M et al (2021) Cognitively unimpaired individuals with a low burden of Aβ pathology have a distinct CSF biomarker profile. Alzheimers Res Ther 13:134. 10.1186/s13195-021-00863-y 34315519 10.1186/s13195-021-00863-y PMC8314554

[32] Thijssen EH et al (2020) Diagnostic value of plasma phosphorylated tau181 in Alzheimer’s disease and frontotemporal lobar degeneration. Nat Med 26(3):387–397. 10.1038/s41591-020-0762-2 32123386 10.1038/s41591-020-0762-2 PMC7101073

[33] Salvadó G et al (2019) Centiloid cut-off values for optimal agreement between PET and CSF core AD biomarkers. Alz Res Ther 11(1):27. 10.1186/s13195-019-0478-z 10.1186/s13195-019-0478-z PMC6429814 30902090

[34] Hu Y et al (2024) Fluid biomarkers in the context of amyloid-targeting disease-modifying treatments in Alzheimer’s disease. Med 5(10):1206–1226. 10.1016/j.medj.2024.08.004 39255800 10.1016/j.medj.2024.08.004

[35] Mielke MM, Fowler NR (2024) Alzheimer disease blood biomarkers: considerations for population-level use. Nat Rev Neurol 20(8):495–504. 10.1038/s41582-024-00989-1 38862788 10.1038/s41582-024-00989-1 PMC11347965

[36] Kac PR et al (2024) Plasma p-tau212 as a biomarker of sporadic and Down Syndrome Alzheimers disease. medRxiv. 10.1101/2024.10.31.24316469.

[37] Cummings J, Zhou Y, Lee G, Zhong K, Fonseca J, Cheng F (2024) Alzheimer’s disease drug development pipeline: 2024. Alzheimer’s Dement: Trans Res Clin Interv 10(2):e12465. 10.1002/trc2.12465 10.1002/trc2.12465 PMC11040692 38659717

[38] Rahman A, Grundke-Iqbal I, Iqbal K (2005) Phosphothreonine-212 of Alzheimer abnormally hyperphosphorylated tau is a preferred substrate of protein phosphatase-1. Neurochem Res 30(2):277–287. 10.1007/s11064-005-2483-9 15895832 10.1007/s11064-005-2483-9

[39] Woods YL et al (2001) The kinase DYRK phosphorylates protein-synthesis initiation factor eIF2Bepsilon at Ser539 and the microtubule-associated protein tau at Thr212: potential role for DYRK as a glycogen synthase kinase 3-priming kinase. Biochem J 355(Pt 3):609–615. 10.1042/bj3550609 11311121 10.1042/bj3550609 PMC1221774

[40] Li Z, Yin B, Zhang S, Lan Z, Zhang L (2023) Targeting protein kinases for the treatment of Alzheimer’s disease: recent progress and future perspectives. Eur J Med Chem 261:115817. 10.1016/j.ejmech.2023.115817 37722288 10.1016/j.ejmech.2023.115817

[41] Liu F et al (2008) Overexpression of Dyrk1A contributes to neurofibrillary degeneration in Down syndrome. FASEB J 22(9):3224–3233. 10.1096/fj.07-104539 18509201 10.1096/fj.07-104539 PMC2518253

[42] Naert G et al (2015) Leucettine L41, a DYRK1A-preferential DYRKs/CLKs inhibitor, prevents memory impairments and neurotoxicity induced by oligomeric Aβ25–35 peptide administration in mice. Eur Neuropsychopharmacol 25(11):2170–2182. 10.1016/j.euroneuro.2015.03.018 26381812 10.1016/j.euroneuro.2015.03.018

[43] Nguyen TL et al (2018) Correction of cognitive deficits in mouse models of Down syndrome by a pharmacological inhibitor of DYRK1A. Dis Model Mech 11(9):dmm035634. 10.1242/dmm.035634 30115750 10.1242/dmm.035634 PMC6176987

[44] Gehlot P, Pathak R, Kumar S, Choudhary NK, Vyas VK (2024) A review on synthetic inhibitors of dual-specific tyrosine phosphorylation-regulated kinase 1A (DYRK1A) for the treatment of Alzheimer’s disease (AD). Bioorg Med Chem 113:117925. 10.1016/j.bmc.2024.117925 39357433 10.1016/j.bmc.2024.117925

[45] Chen H et al (2024) Discovery of ZJCK-6-46: a potent, selective, and orally available dual-specificity tyrosine phosphorylation-regulated kinase 1A inhibitor for the treatment of Alzheimer’s disease. J Med Chem 67(15):12571–12600. 10.1021/acs.jmedchem.4c00483 39041662 10.1021/acs.jmedchem.4c00483

[46] Lindberg MF et al (2023) Comparative efficacy and selectivity of pharmacological inhibitors of DYRK and CLK protein kinases. J Med Chem 66(6):4106–4130. 10.1021/acs.jmedchem.2c02068 36876904 10.1021/acs.jmedchem.2c02068

[47] Lindberg MF et al (2023) Chemical, biochemical, cellular, and physiological characterization of Leucettinib-21, a Down syndrome and Alzheimer’s disease drug candidate. J Med Chem 66(23):15648–15670. 10.1021/acs.jmedchem.3c01888 38051674 10.1021/acs.jmedchem.3c01888

[48] Meijer L et al (2024) Leucettinib-21, a DYRK1A kinase inhibitor as clinical drug candidate for Alzheimer’s disease and Down syndrome. J Alzheimers Dis 101(s1):S95–S113. 10.3233/JAD-240078 39422950 10.3233/JAD-240078

[49] Triana‐Baltzer G et al (2021) Development and validation of a high‐sensitivity assay for measuring p217+tau in plasma. Alzheimers Dement Diagn Assess Dis Monit 13(1):e12204. 10.1002/dad2.12204 10.1002/dad2.12204 PMC8158165 34095436

[50] Janelidze S et al (2024) Plasma phosphorylated tau 217 and Aβ42/40 to predict early brain Aβ accumulation in people without cognitive impairment. JAMA Neurol 81(9):947–957. 10.1001/jamaneurol.2024.2619 39068669 10.1001/jamaneurol.2024.2619 PMC11284634

[51] Ashton NJ et al (2024) Diagnostic accuracy of a plasma phosphorylated tau 217 immunoassay for Alzheimer disease pathology. JAMA Neurol 81(3):255–263. 10.1001/jamaneurol.2023.5319 38252443 10.1001/jamaneurol.2023.5319 PMC10804282

[52] Mattsson-Carlgren N et al (2024) Plasma biomarker strategy for selecting patients with Alzheimer disease for antiamyloid immunotherapies. JAMA Neurol 81(1):69–78. 10.1001/jamaneurol.2023.4596 38048096 10.1001/jamaneurol.2023.4596 PMC10696515

