# Supplementary material for: Plasma p-tau212 identifies cognitively unimpaired individuals with emerging amyloid-β pathology
Source: J Neurol. 2025 Dec 25;273(1):48. doi: 10.1007/s00415-025-13572-5 (PMC12740965; doi:10.1007/s00415-025-13572-5)
Supplement: Supplementary file 1 — Supplementary file1 (DOCX 3741 KB) [file 415_2025_13572_MOESM1_ESM.docx]

**Supplementary Table 1.** Demographic table of the study cohort stratified by Aβ PET status (A+ > 24 Centiloids)

| **Characteristic** | **N** | **PET A- N = 251** | **PET A+ N = 26** | **p-value** |
| --- | --- | --- | --- | --- |
| **Age, Median (Q1, Q3)** | 277 | 60.2 (57.4, 64.1) | 65.0 (61.6, 66.1) | <0.001*^1^* |
| **Sex, n (%)** | 277 |  |  | 0.3*^2^* |
| Men |  | 101 (40%) | 13 (50%) |  |
| Women |  | 150 (60%) | 13 (50%) |  |
| **APOE_carriership, n (%)** | 277 |  |  | 0.059*^2^* |
| Non_carrier |  | 116 (46%) | 7 (27%) |  |
| Carrier |  | 135 (54%) | 19 (73%) |  |
| **MMSE, Median (Q1, Q3)** | 277 | 30.00 (29.00, 30.00) | 29.00 (29.00, 30.00) | 0.7*^1^* |
| **BMI, Median (Q1, Q3)** | 277 | 26.2 (23.8, 29.8) | 26.9 (25.5, 28.3) | 0.9*^1^* |
| **Estimated GFR, Median (Q1, Q3)** | 275 | 93 (79, 108) | 88 (71, 104) | 0.3*^1^* |
| **amyloid PET Centiloids, Median (Q1, Q3)** | 277 | -3 (-8, 2) | 40 (27, 53) | <0.001*^1^* |
| **CSF Aβ42 (pg/mL), Median (Q1, Q3)** | 263 | 1,277 (954, 1,662) | 735 (623, 819) | <0.001*^1^* |
| **CSF Aβ42/40, Median (Q1, Q3)** | 263 | 0.082 (0.068, 0.090) | 0.044 (0.035, 0.048) | <0.001*^1^* |
| **CSF t-tau (pg/mL), Median (Q1, Q3)** | 263 | 176 (145, 217) | 238 (194, 276) | <0.001*^1^* |
| **CSF p-tau181 (pg/mL), Median (Q1, Q3)** | 263 | 14.0 (11.0, 17.9) | 21.4 (16.0, 24.9) | <0.001*^1^* |
| **Plasma p-tau212 (pg/mL), Median (Q1, Q3)** | 277 | 0.11 (0.07, 0.17) | 0.28 (0.18, 0.37) | <0.001*^1^* |
| **Plasma p-tau217 (pg/mL), Median (Q1, Q3)** | 277 | 0.13 (0.10, 0.16) | 0.23 (0.19, 0.29) | <0.001*^1^* |
| **Plasma p-tau181 (pg/mL), Median (Q1, Q3)** | 277 | 8.55 (7.05, 10.77) | 11.05 (9.64, 13.05) | <0.001^1^ |
| **Plasma p-tau231 (pg/mL), Median (Q1, Q3)** | 277 | 9.9 (7.4, 13.6) | 13.8 (10.0, 19.2) | <0.001^1^ |

Numerical variables are presented as median and interquartile range (IQR: Q1–Q3), while categorical variables are shown as counts and percentages (%). Differences in numerical variables between CSF-defined Aβ groups were assessed using the Wilcoxon rank-sum test. For categorical variables, group differences were evaluated using Pearson’s chi-squared (χ²) test. Participants are classified based on their CSF Aβ status (Aβ-positive defined as Aβ > 24 Centiloids.)

Abbreviations: Aβ: amyloid beta; *APOE*4: Apolipoprotein E4; BMI: Body Mass Index; CSF: Cerebrospinal Fluid; eGFR: estimated Glomerular Filtration Rate; MMSE: Mini–Mental State Examination; p-tauX: tau phosphorylated at amino acid X; PET: Positron Emission Tomography; t-tau: total-tau.

*^1^Wilcoxon rank sum test ^2^Pearson's Chi-squared test*

**Supplementary Table 2.** Demographic table of the study cohort stratified by AT status (A+ - Aβ42/40 < 0.071 T+ > 24 pg/mL)

| **Characteristic** | **N** | **CSF A-T- N = 189** | **CSF A+T- N = 85** | **CSF A+T+ N = 20** | **CSF A-T+ N = 9** | **p-value** |
| --- | --- | --- | --- | --- | --- | --- |
| **Age, Median (Q1, Q3)** | 303 | 60.2 (57.6, 63.9) | 62.5 (58.3, 65.3) | 63.8 (59.8, 66.7) | 62.9 (60.4, 64.2) | 0.040*^1^* |
| **Sex, n (%)** | 303 |  |  |  |  | 0.7*2* |
| Men |  | 74 (39%) | 39 (46%) | 8 (40%) | 3 (33%) |  |
| Women |  | 115 (61%) | 46 (54%) | 12 (60%) | 6 (67%) |  |
| **APOE_carriership, n (%)** | 303 |  |  |  |  | <0.001*^2^* |
| Non_carrier |  | 107 (57%) | 17 (20%) | 7 (35%) | 7 (78%) |  |
| Carrier |  | 82 (43%) | 68 (80%) | 13 (65%) | 2 (22%) |  |
| **MMSE, Median (Q1, Q3)** | 303 | 29.00 (29.00, 30.00) | 30.00 (29.00, 30.00) | 29.00 (28.00, 30.00) | 30.00 (29.00, 30.00) | 0.6*^1^* |
| **BMI, Median (Q1, Q3)** | 303 | 26.4 (24.4, 29.9) | 26.1 (23.9, 29.1) | 25.9 (24.4, 28.6) | 24.5 (22.6, 28.0) | 0.5*^1^* |
| **eGFR, Median (Q1, Q3)** | 301 | 93 (79, 109) | 94 (75, 106) | 81 (71, 103) | 91 (71, 107) | 0.3*^1^* |
| **amyloid-PET Centiloids, Median (Q1, Q3)** | 263 | -5 (-9, 0) | 9 (0, 20) | 26 (-1, 39) | -4 (-7, -2) | <0.001*^1^* |
| **CSF Aβ42 (pg/mL), Median (Q1, Q3)** | 303 | 1,383 (1,099, 1,819) | 825 (697, 1,021) | 801 (709, 1,296) | 2,461 (2,103, 2,620) | <0.001*^1^* |
| **CSF Aβ42/40 , Median (Q1, Q3)** | 303 | 0.085 (0.081, 0.093) | 0.055 (0.044, 0.064) | 0.041 (0.031, 0.053) | 0.095 (0.074, 0.099) | <0.001*^1^* |
| **CSF t-tau (pg/mL), Median (Q1, Q3)** | 303 | 169 (142, 206) | 185 (156, 219) | 315 (299, 371) | 298 (287, 314) | <0.001*^1^* |
| **CSF p-tau181 (pg/mL), Median (Q1, Q3)** | 303 | 13.4 (10.6, 17.0) | 14.8 (12.4, 18.4) | 28.6 (25.6, 34.8) | 24.8 (24.3, 27.6) | <0.001*^1^* |
| **Plasma p-tau212 (pg/mL), Median (Q1, Q3)** | 303 | 0.10 (0.06, 0.16) | 0.14 (0.09, 0.26) | 0.28 (0.18, 0.36) | 0.10 (0.10, 0.12) | <0.001*^1^* |
| **Plasma p-tau217 (pg/mL), Median (Q1, Q3)** | 303 | 0.13 (0.10, 0.16) | 0.15 (0.12, 0.21) | 0.21 (0.15, 0.28) | 0.12 (0.09, 0.15) | <0.001*^1^* |
| **Plasma p-tau181 (pg/mL), Median (Q1, Q3)** | 303 | 8.11 (6.87, 10.00) | 9.52 (7.76, 11.40) | 10.93 (10.00, 12.09) | 8.55 (7.28, 11.55) | <0.001*^1^* |
| **Plasma p-tau231 (pg/mL), Median (Q1, Q3)** | 303 | 9.0 (7.1, 11.6) | 11.2 (8.7, 15.2) | 17.3 (12.1, 22.5) | 11.3 (9.4, 12.2) | <0.001*^1^* |

Numerical variables are presented as median and interquartile range (IQR: Q1–Q3), while categorical variables are shown as counts and percentages (%). Differences in numerical variables between CSF-defined Aβ groups were assessed using the Wilcoxon rank-sum test. For categorical variables, group differences were evaluated using Pearson’s chi-squared (χ²) test. Participants are classified based on their CSF Aβ status (Aβ-positive defined as Aβ > 24 Centiloids.)

Abbreviations: Aβ: amyloid beta; *APOE*4: Apolipoprotein E4; BMI: Body Mass Index; CSF: Cerebrospinal Fluid; eGFR: estimated Glomerular Filtration Rate; MMSE: Mini–Mental State Examination; p-tauX: tau phosphorylated at amino acid X; PET: Positron Emission Tomography; t-tau: total-tau.

*^1^Wilcoxon rank sum test ^2^Pearson's Chi-squared test*

**Supplementary Table 3.** Demographic table for the amyloid burden status (CSF A+ - Aβ42/40 < 0.071, PET A+ > 30 Centiloids)

| **Characteristic** | **N** | **Negative N = 173** | **Low amyloid burden N = 74** | **Positive N = 16** | **p-value** |
| --- | --- | --- | --- | --- | --- |
| **Age, Median (Q1, Q3)** | 263 | 60.2 (57.7, 63.9) | 60.8 (58.1, 64.9) | 65.8 (63.1, 66.9) | <0.001*^1^* |
| **Sex, n (%)** | 263 |  |  |  | 0.3*^2^* |
| Men |  | 64 (37%) | 35 (47%) | 7 (44%) |  |
| Women |  | 109 (63%) | 39 (53%) | 9 (56%) |  |
| **APOE_carriership, n (%)** | 263 |  |  |  | <0.001*^2^* |
| Non_carrier |  | 101 (58%) | 14 (19%) | 5 (31%) |  |
| Carrier |  | 72 (42%) | 60 (81%) | 11 (69%) |  |
| **MMSE, Median (Q1, Q3)** | 263 | 29.00 (29.00, 30.00) | 30.00 (29.00, 30.00) | 29.00 (28.00, 30.00) | 0.057*^1^* |
| **BMI, Median (Q1, Q3)** | 263 | 26.4 (24.3, 29.8) | 26.1 (23.7, 29.3) | 26.2 (25.4, 27.4) | 0.8*^1^* |
| **eGFR, Median (Q1, Q3)** | 261 | 93 (79, 109) | 94 (75, 107) | 85 (70, 100) | 0.2*^1^* |
| **amyloid PET Centiloids, Median (Q1, Q3)** | 263 | -4 (-9, 0) | 7 (-2, 16) | 46 (38, 53) | <0.001*^1^* |
| **CSF Aβ42 (pg/mL), Median (Q1, Q3)** | 263 | 1,398 (1,145, 1,892) | 864 (751, 1,064) | 714 (623, 862) | <0.001*^1^* |
| **CSF Aβ42/40, Median (Q1, Q3)** | 263 | 0.086 (0.081, 0.093) | 0.057 (0.047, 0.064) | 0.043 (0.033, 0.046) | <0.001*^1^* |
| **CSF t-tau (pg/mL), Median (Q1, Q3)** | 263 | 173 (143, 214) | 188 (156, 228) | 260 (221, 297) | <0.001*^1^* |
| **CSF p-tau181 (pg/mL), Median (Q1, Q3)** | 263 | 13.8 (10.8, 17.7) | 14.8 (12.5, 18.7) | 23.6 (20.3, 26.8) | <0.001*^1^* |
| **Plasma p-tau212 (pg/mL), Median (Q1, Q3)** | 263 | 0.10 (0.06, 0.15) | 0.14 (0.08, 0.25) | 0.27 (0.16, 0.37) | <0.001*^1^* |
| **Plasma p-tau217 (pg/mL), Median (Q1, Q3)** | 263 | 0.12 (0.10, 0.15) | 0.14 (0.11, 0.19) | 0.26 (0.21, 0.29) | <0.001*^1^* |
| **Plasma p-tau181 (pg/mL), Median (Q1, Q3)** | 263 | 8.26 (6.87, 10.38) | 9.86 (7.79, 11.78) | 10.98 (9.77, 12.14) | <0.001^1^ |
| **Plasma p-tau231 (pg/mL), Median (Q1, Q3)** | 263 | 9.3 (7.1, 11.9) | 12.6 (9.8, 15.3) | 17.3 (9.0, 22.5) | <0.001^1^ |

Numerical variables are presented as median and interquartile range (IQR: Q1–Q3), while categorical variables are shown as counts and percentages (%). Differences in numerical variables between CSF-defined Aβ groups were assessed using the Wilcoxon rank-sum test. For categorical variables, group differences were evaluated using Pearson’s chi-squared (χ²) test. Participants are classified based on their CSF Aβ status (Aβ-positive defined as Aβ > 24 Centiloids.)

Abbreviations: Aβ: amyloid beta; *APOE*4: Apolipoprotein E4; BMI: Body Mass Index; CSF: Cerebrospinal Fluid; eGFR: estimated Glomerular Filtration Rate; MMSE: Mini–Mental State Examination; p-tauX: tau phosphorylated at amino acid X; PET: Positron Emission Tomography; t-tau: total-tau.

*^1^Kruskal-Wallis rank sum test, ^2^Fisher’s exact test*


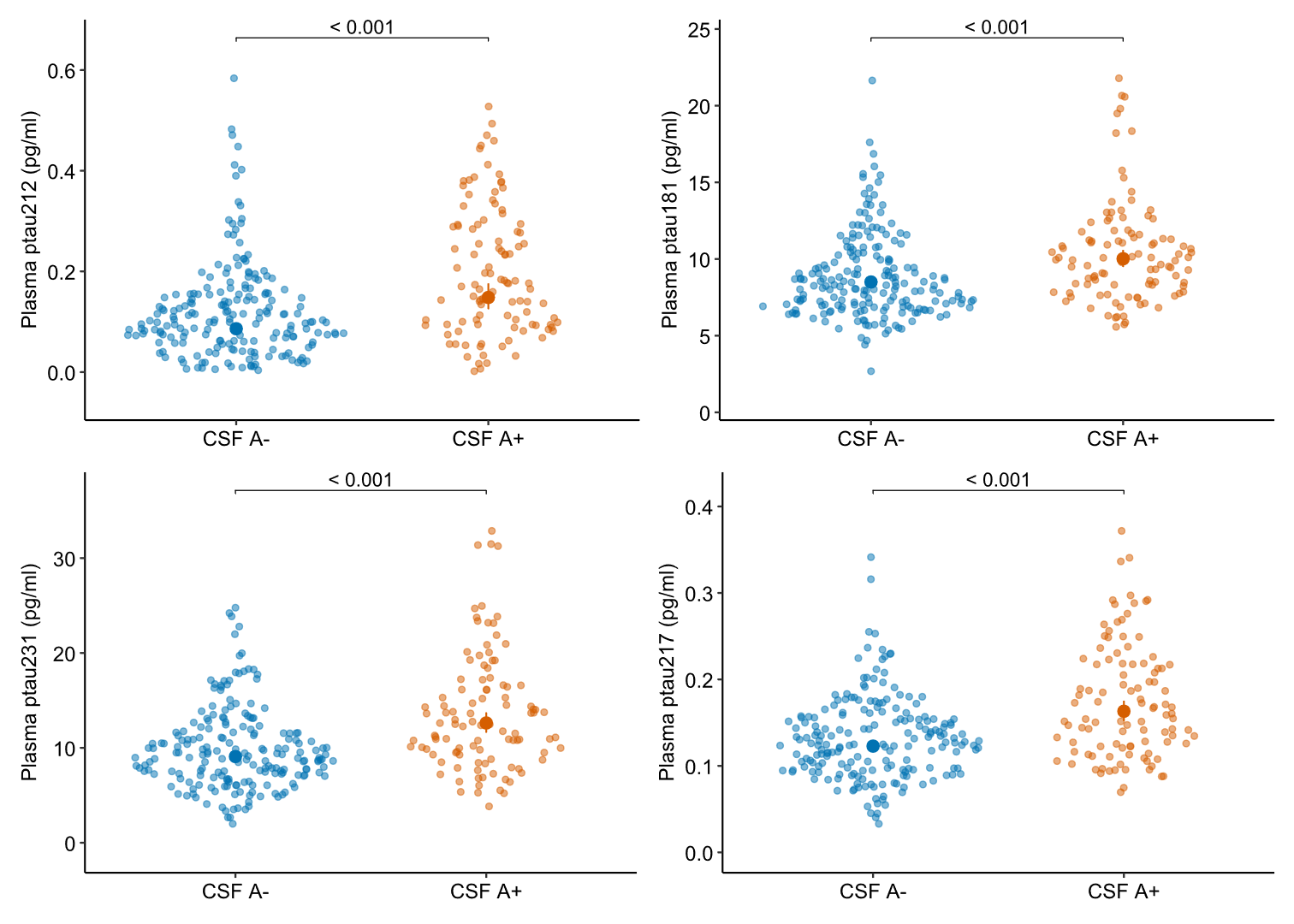


**Supplementary Fig. 1 Levels of adjusted plasma p-tau biomarkers in cognitively unimpaired participants respectively to their CSF amyloid status**

The figure shows plasma levels of the plasma biomarkers in participants classified according to their CSF amyloid status (CSF A+ - Aβ42/40 < 0.071). N=198 cognitively unimpaired (CU) participants were classified as negative; n=105 CU participants were classified as positive. Plots show the original data points as well as the estimated marginal means (dots) from a linear model adjusted by age, sex, Body Mass Index (BMI) and estimated Glomerular Filtration Rate (eGFR). The lines adjacent to the dots represent the 95% percent confidence interval of the estimated marginal mean, while the p values represent the pairwise contrasts of the different amyloid subgroups.


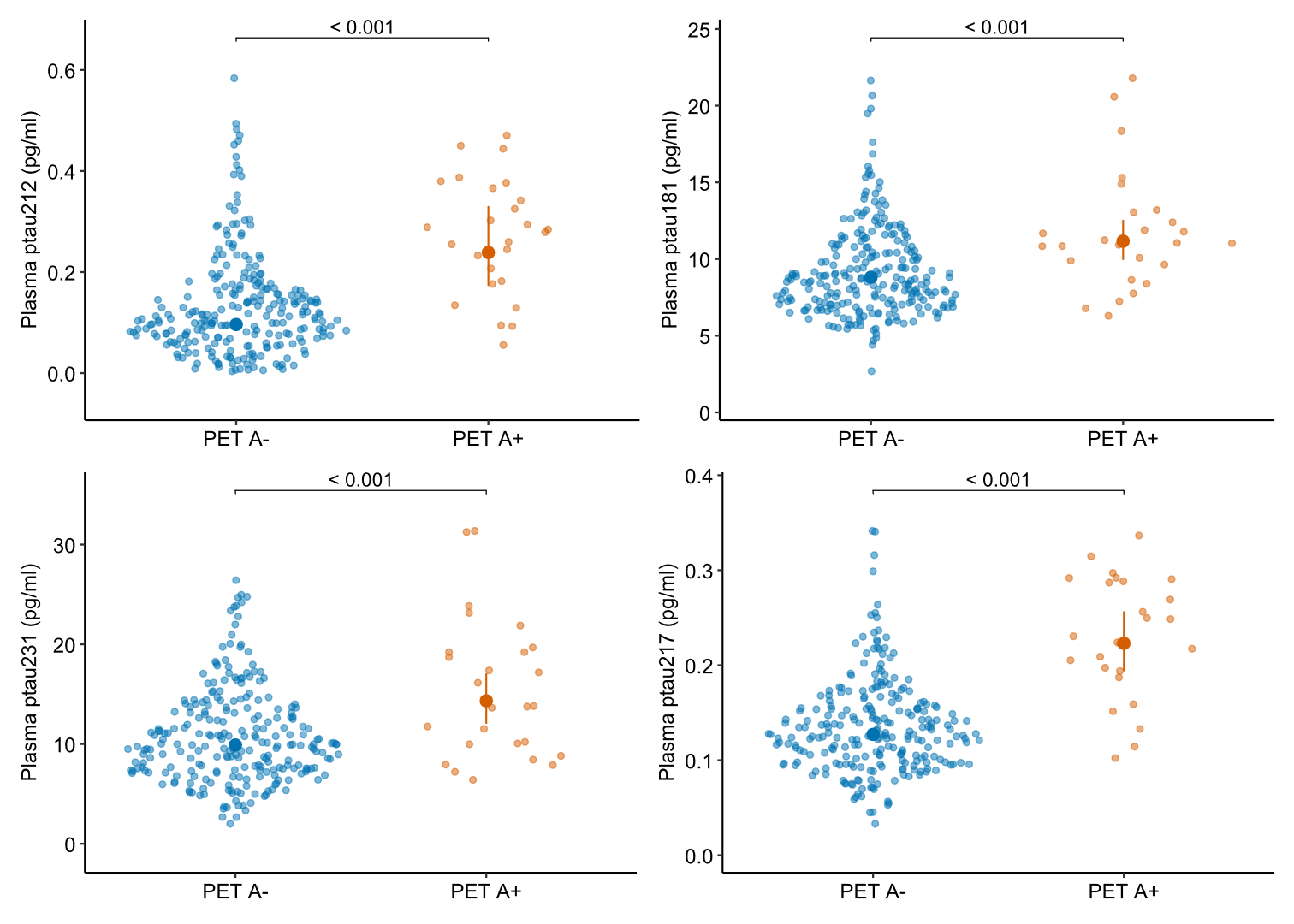


**Supplementary Fig. 2 Levels of adjusted plasma p-tau biomarkers in cognitively unimpaired participants respectively to their PET amyloid status**

The figure shows plasma levels of the plasma biomarkers in participants classified according to their PET amyloid status (PET A+ > 24 Centiloids). N=251 cognitively unimpaired (CU) participants were classified as negative; n=26 CU participants were classified as positive. Plots show the original data points as well as the estimated marginal means (dots) from a linear model adjusted by age, sex, Body Mass Index (BMI) and estimated Glomerular Filtration Rate (eGFR). The lines adjacent to the dots represent the 95% percent confidence interval of the estimated marginal mean, while the p values represent the pairwise contrasts of the different amyloid subgroups.


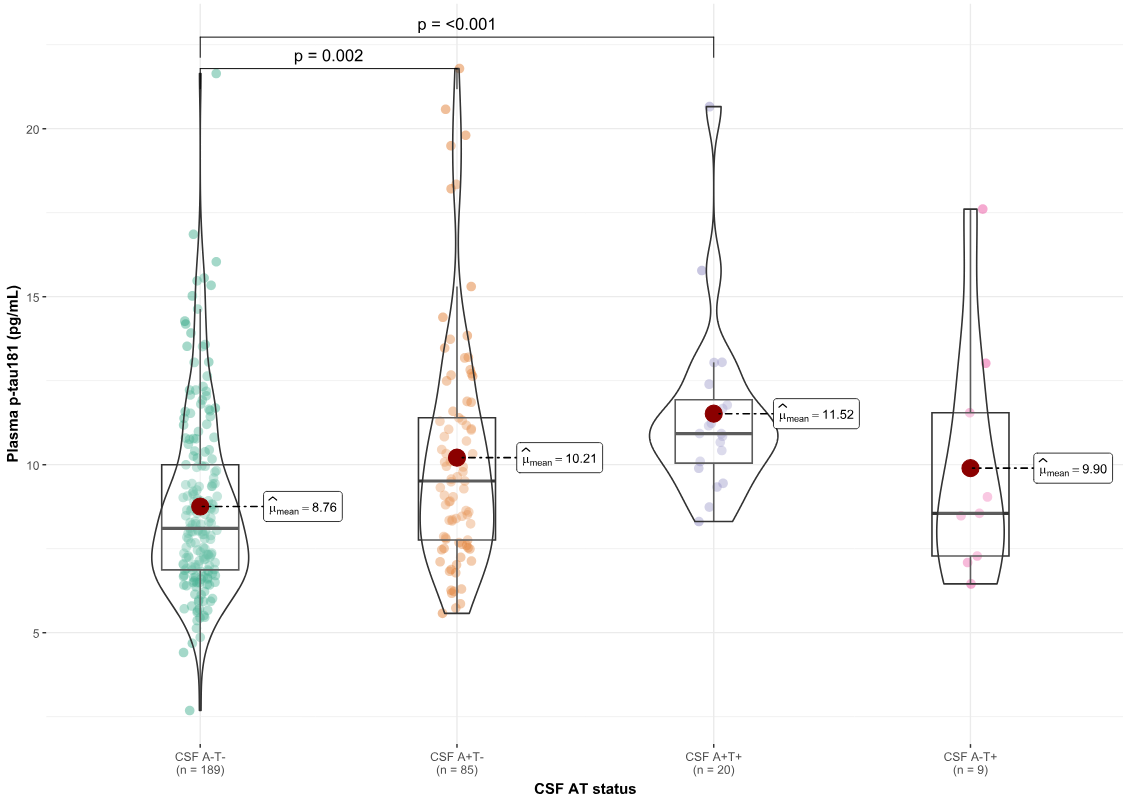


**Supplementary Fig. 3 Levels of plasma p-tau181 in cognitively unimpaired participants respectively to their CSF AT status**

The figure shows plasma levels of the plasma biomarkers in participants classified according to their CSF amyloid and tau (T_1_) status (Aβ42/40 <0.071; T+ >24 pg/ml). N=189 cognitively unimpaired (CU) participants were classified as A-T-; n=85 CU participants were classified as A+T-; n=20 CU participants were classified as A+T+; n=9 CU participants were classified as A-T+. Boxplots included in the violin plots are shown as a median and interquartile range (IQR), upper whisker is 75^th^ percentile plus 1.5 times IQR and lower whisker is 25^th^ percentile minus 1.5 IQR. Red dot represents mean of the group. Group differences were examined using Dwass-Steel-Critchlow-Fligner test.


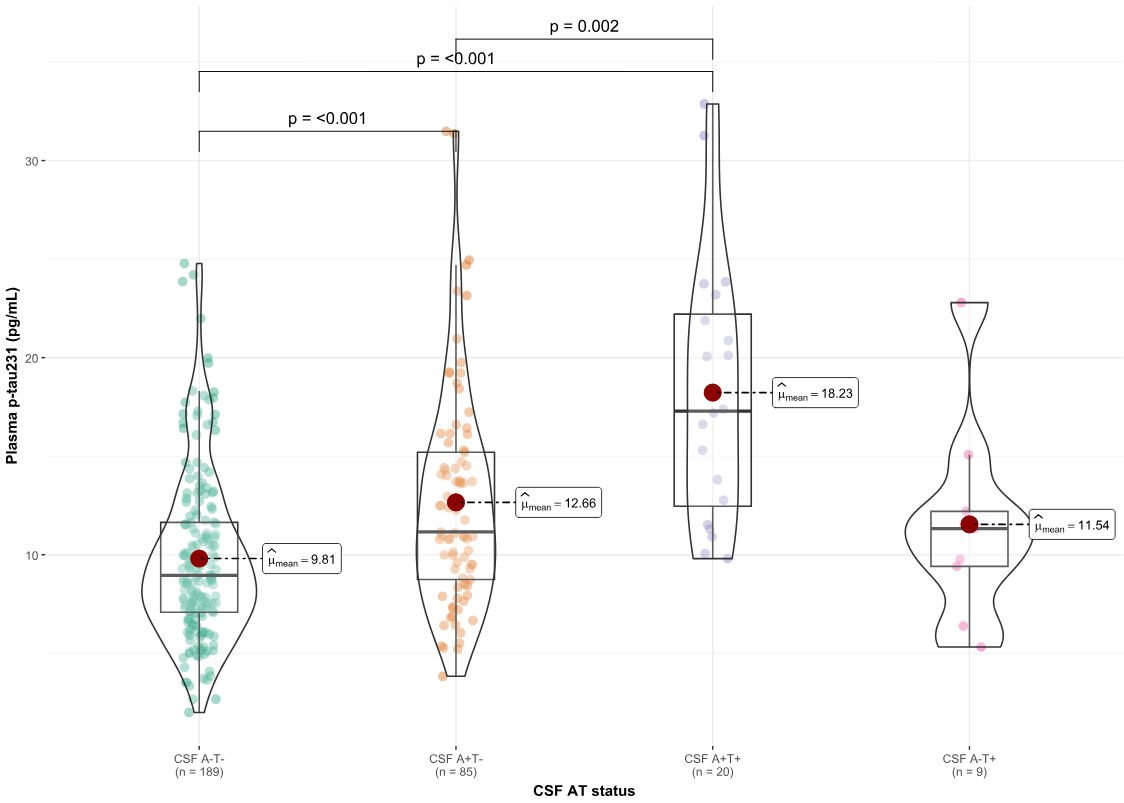


**Supplementary Fig. 4 Levels of plasma p-tau231 in cognitively unimpaired participants respectively to their CSF AT status**

The figure shows plasma levels of the plasma biomarkers in participants classified according to their CSF amyloid and tau (T_1_) status (Aβ42/40 <0.071; T+ >24 pg/ml). N=189 cognitively unimpaired (CU) participants were classified as A-T-; n=85 CU participants were classified as A+T-; n=20 CU participants were classified as A+T+; n=9 CU participants were classified as A-T+. Boxplots included in the violin plots are shown as a median and interquartile range (IQR), upper whisker is 75^th^ percentile plus 1.5 times IQR and lower whisker is 25^th^ percentile minus 1.5 IQR. Red dot represents mean of the group. Group differences were examined using Dwass-Steel-Critchlow-Fligner test.


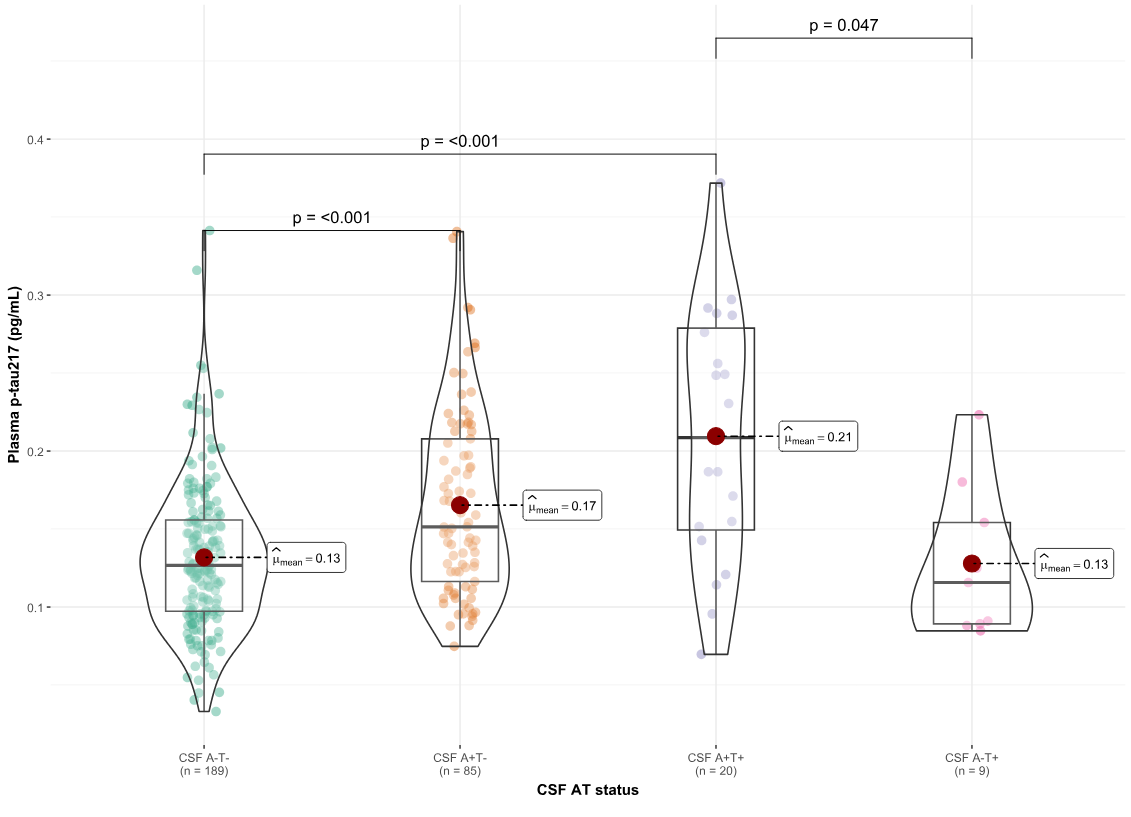


**Supplementary Fig. 5 Levels of plasma p-tau217 in cognitively unimpaired participants respectively to their CSF AT status**

The figure shows plasma levels of the plasma biomarkers in participants classified according to their CSF amyloid and tau (T_1_) status (Aβ42/40 <0.071; T+ >24 pg/ml). N=189 cognitively unimpaired (CU) participants were classified as A-T-; n=85 CU participants were classified as A+T-; n=20 CU participants were classified as A+T+; n=9 CU participants were classified as A-T+. Boxplots included in the violin plots are shown as a median and interquartile range (IQR), upper whisker is 75^th^ percentile plus 1.5 times IQR and lower whisker is 25^th^ percentile minus 1.5 IQR. Red dot represents mean of the group. Group differences were examined using Dwass-Steel-Critchlow-Fligner test.


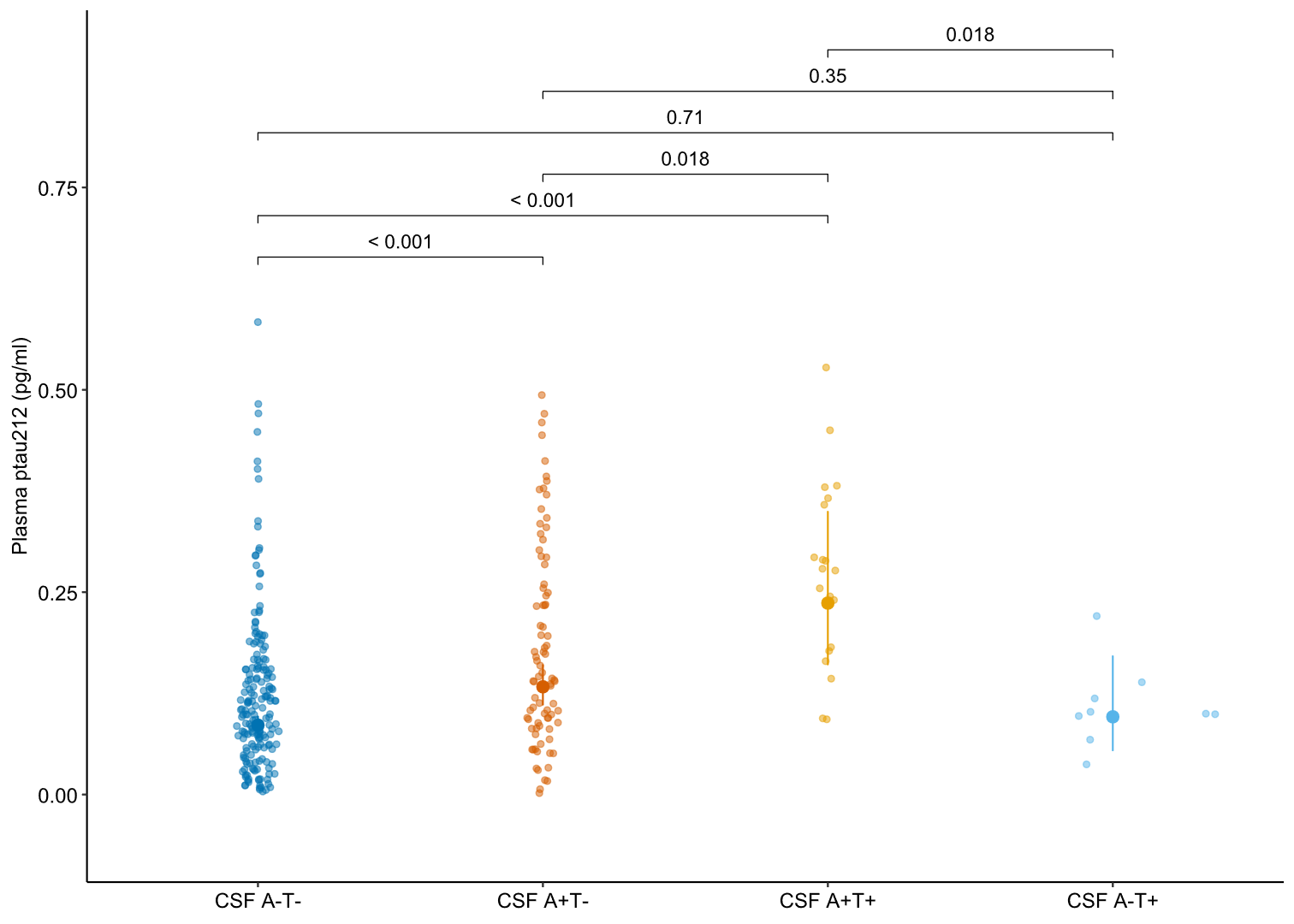


**Supplementary Fig. 6 Levels of adjusted plasma p-tau212 in cognitively unimpaired participants respectively to their CSF AT status**

The figure shows plasma levels of the plasma biomarkers in participants classified according to their CSF amyloid and tau (T_1_) status (Aβ42/40 <0.071; T+ >24 pg/ml). N=189 cognitively unimpaired (CU) participants were classified as A-T-; n=85 CU participants were classified as A+T-; n=20 CU participants were classified as A+T+; n=9 CU participants were classified as A-T+. Plots show the original data points as well as the estimated marginal means (dots) from a linear model adjusted by age, sex, Body Mass Index (BMI) and estimated Glomerular Filtration Rate (eGFR). The lines adjacent to the dots represent the 95% percent confidence interval of the estimated marginal mean, while the p values represent the pairwise contrasts of the different amyloid subgroups.


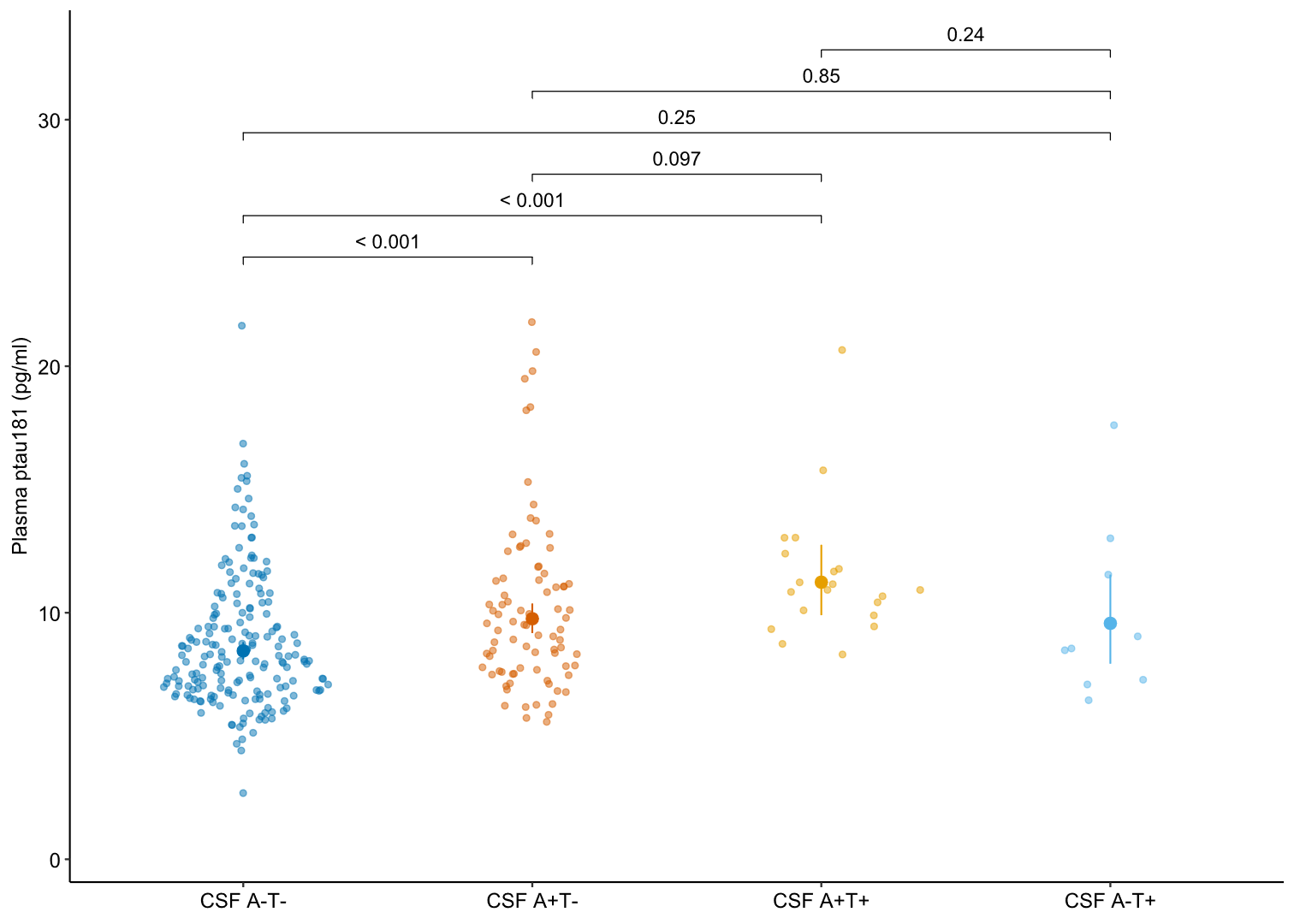


**Supplementary Fig. 7 Levels of adjusted plasma p-tau181 in cognitively unimpaired participants respectively to their CSF AT status**

The figure shows plasma levels of the plasma biomarkers in participants classified according to their CSF amyloid and tau (T_1_) status (Aβ42/40 <0.071; T+ >24 pg/ml). N=189 cognitively unimpaired (CU) participants were classified as A-T-; n=85 CU participants were classified as A+T-; n=20 CU participants were classified as A+T+; n=9 CU participants were classified as A-T+. Plots show the original data points as well as the estimated marginal means (dots) from a linear model adjusted by age, sex, Body Mass Index (BMI) and estimated Glomerular Filtration Rate (eGFR). The lines adjacent to the dots represent the 95% percent confidence interval of the estimated marginal mean, while the p values represent the pairwise contrasts of the different amyloid subgroups.


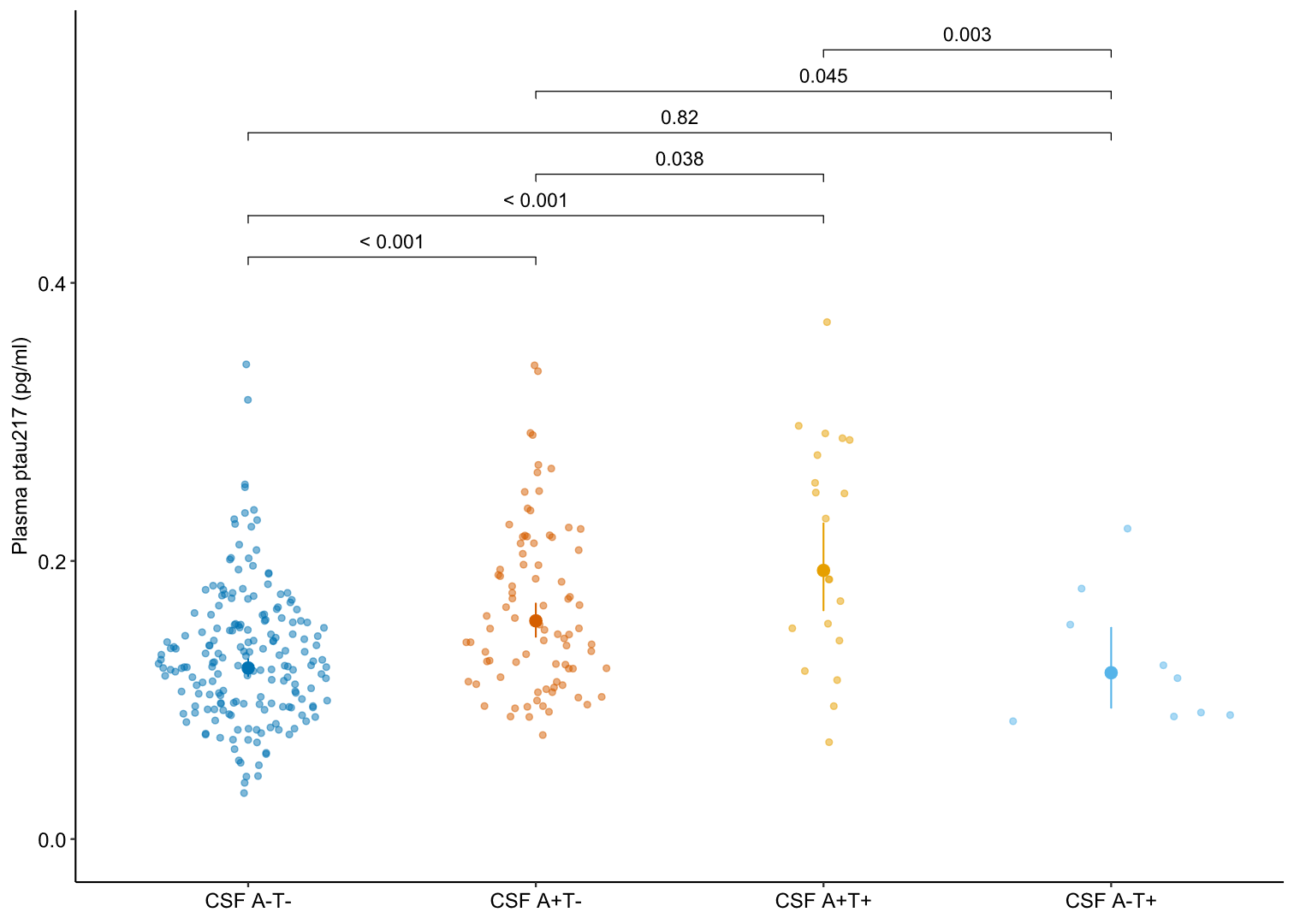


**Supplementary Fig. 8 Levels of adjusted plasma p-tau217 in cognitively unimpaired participants respectively to their CSF AT status**

The figure shows plasma levels of the plasma biomarkers in participants classified according to their CSF amyloid and tau (T_1_) status (Aβ42/40 <0.071; T+ >24 pg/ml). N=189 cognitively unimpaired (CU) participants were classified as A-T-; n=85 CU participants were classified as A+T-; n=20 CU participants were classified as A+T+; n=9 CU participants were classified as A-T+. Plots show the original data points as well as the estimated marginal means (dots) from a linear model adjusted by age, sex, Body Mass Index (BMI) and estimated Glomerular Filtration Rate (eGFR). The lines adjacent to the dots represent the 95% percent confidence interval of the estimated marginal mean, while the p values represent the pairwise contrasts of the different amyloid subgroups.


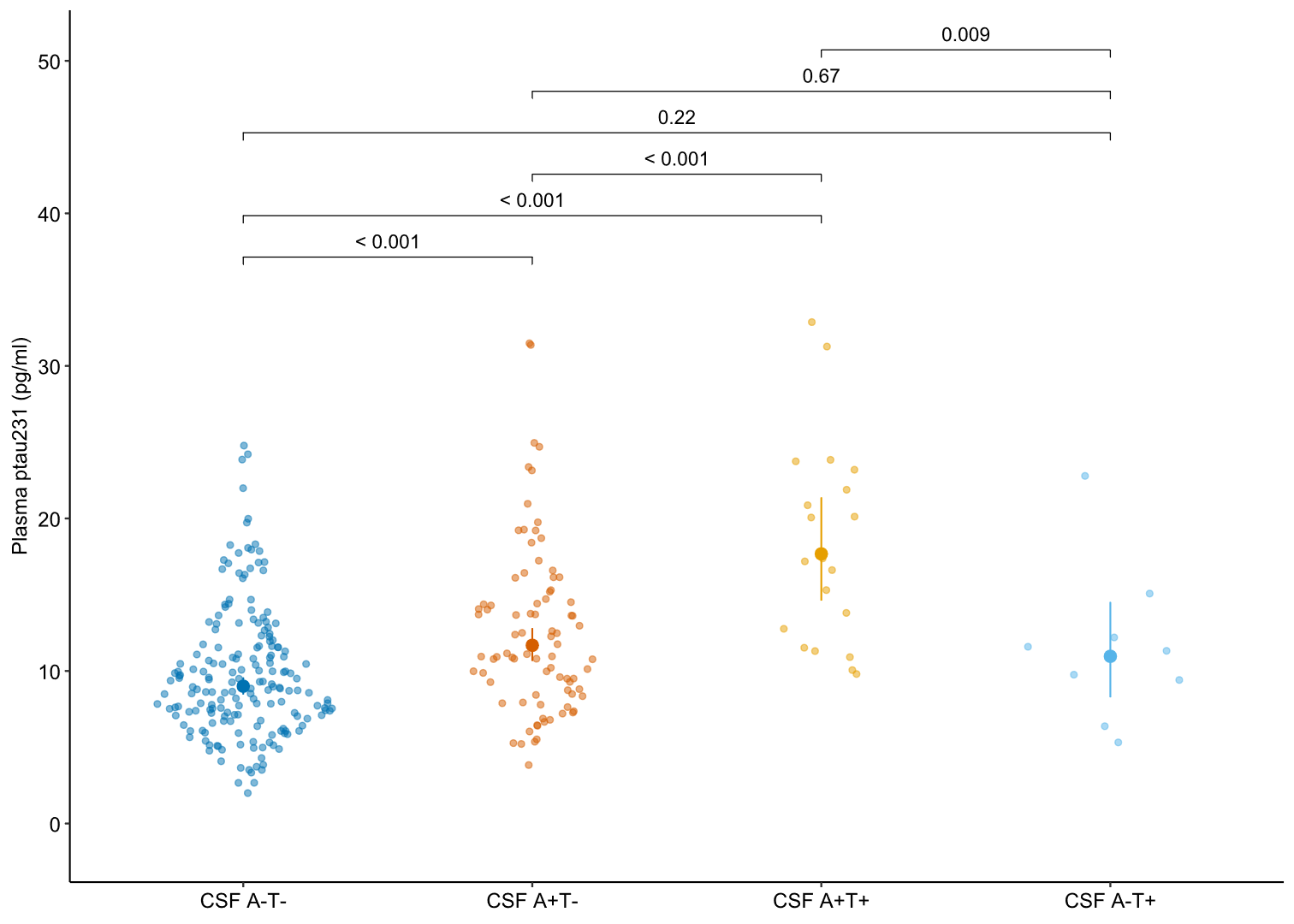


**Supplementary Fig. 9 Levels of adjusted plasma p-tau231 in cognitively unimpaired participants respectively to their CSF AT status**

The figure shows plasma levels of the plasma biomarkers in participants classified according to their CSF amyloid and tau (T_1_) status (Aβ42/40 <0.071; T+ >24 pg/ml). N=189 cognitively unimpaired (CU) participants were classified as A-T-; n=85 CU participants were classified as A+T-; n=20 CU participants were classified as A+T+; n=9 CU participants were classified as A-T+. Plots show the original data points as well as the estimated marginal means (dots) from a linear model adjusted by age, sex, Body Mass Index (BMI) and estimated Glomerular Filtration Rate (eGFR). The lines adjacent to the dots represent the 95% percent confidence interval of the estimated marginal mean, while the pvalues represent the pairwise contrasts of the different amyloid subgroups.

**
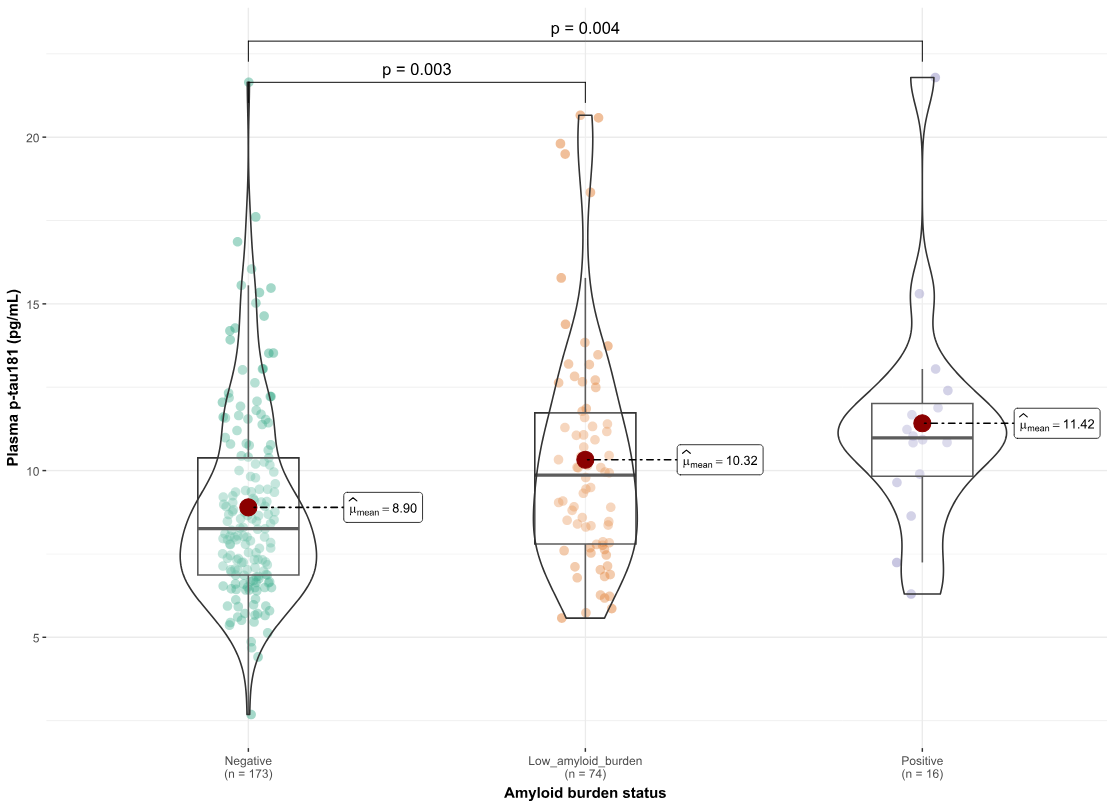
**

**Supplementary Fig. 10 Levels of plasma p-tau181 in cognitively unimpaired participants respectively to their amyloid burden status**

The figure shows plasma levels of the plasma biomarkers in participants classified according to their amyloid burden status (CSF A+ - Aβ42/40 < 0.071, PET A+ > 30 Centiloids). N=173 cognitively unimpaired (CU) participants were classified as negative; n=74 CU participants were classified as low amyloid burden; n=16 CU participants were classified as positive. Boxplots included in the violin plots are shown as a median and interquartile range (IQR), upper whisker is 75^th^ percentile plus 1.5 times IQR and lower whisker is 25^th^ percentile minus 1.5 IQR. Red dot represents mean of the group. Group differences were examined using Dwass-Steel-Critchlow-Fligner test.


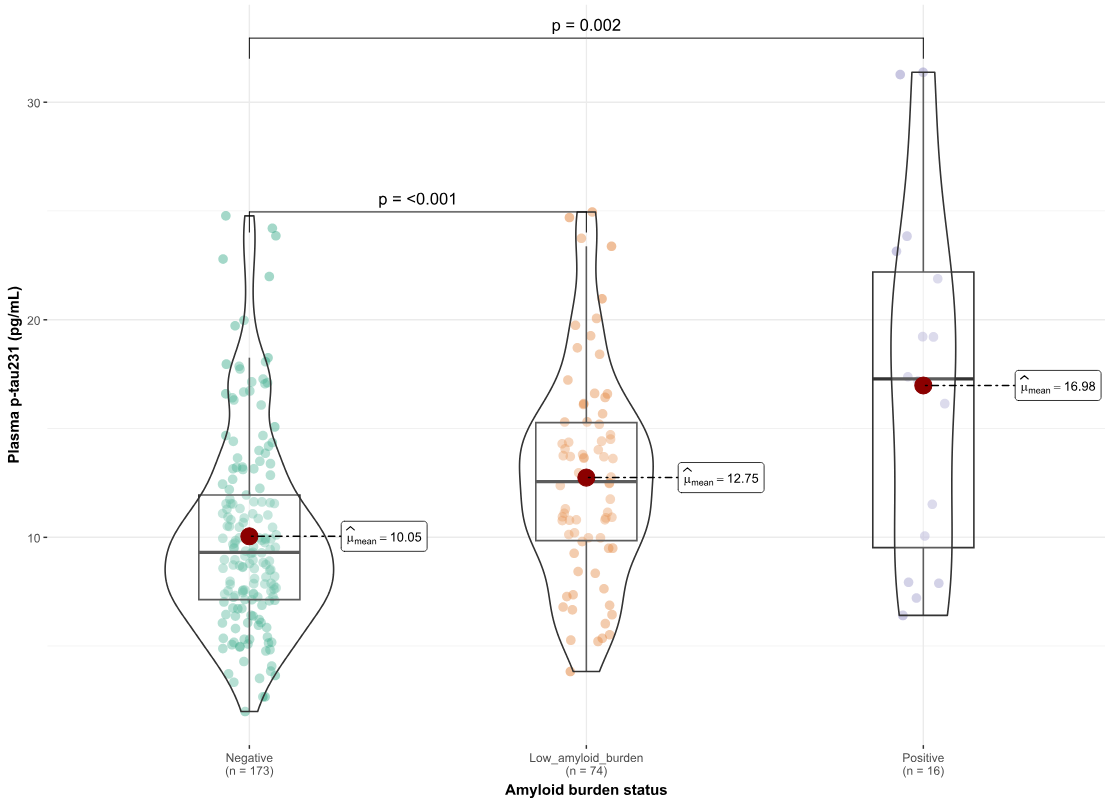


**Supplementary Fig. 11 Levels of plasma p-tau231 in cognitively unimpaired participants respectively to their amyloid burden status**

The figure shows plasma levels of the plasma biomarkers in participants classified according to their amyloid burden status (CSF A+ - Aβ42/40 < 0.071, PET A+ > 30 Centiloids). N=173 cognitively unimpaired (CU) participants were classified as negative; n=74 CU participants were classified as low amyloid burden; n=16 CU participants were classified as positive. Boxplots included in the violin plots are shown as a median and interquartile range (IQR), upper whisker is 75^th^ percentile plus 1.5 times IQR and lower whisker is 25^th^ percentile minus 1.5 IQR. Red dot represents mean of the group. Group differences were examined using Dwass-Steel-Critchlow-Fligner test.


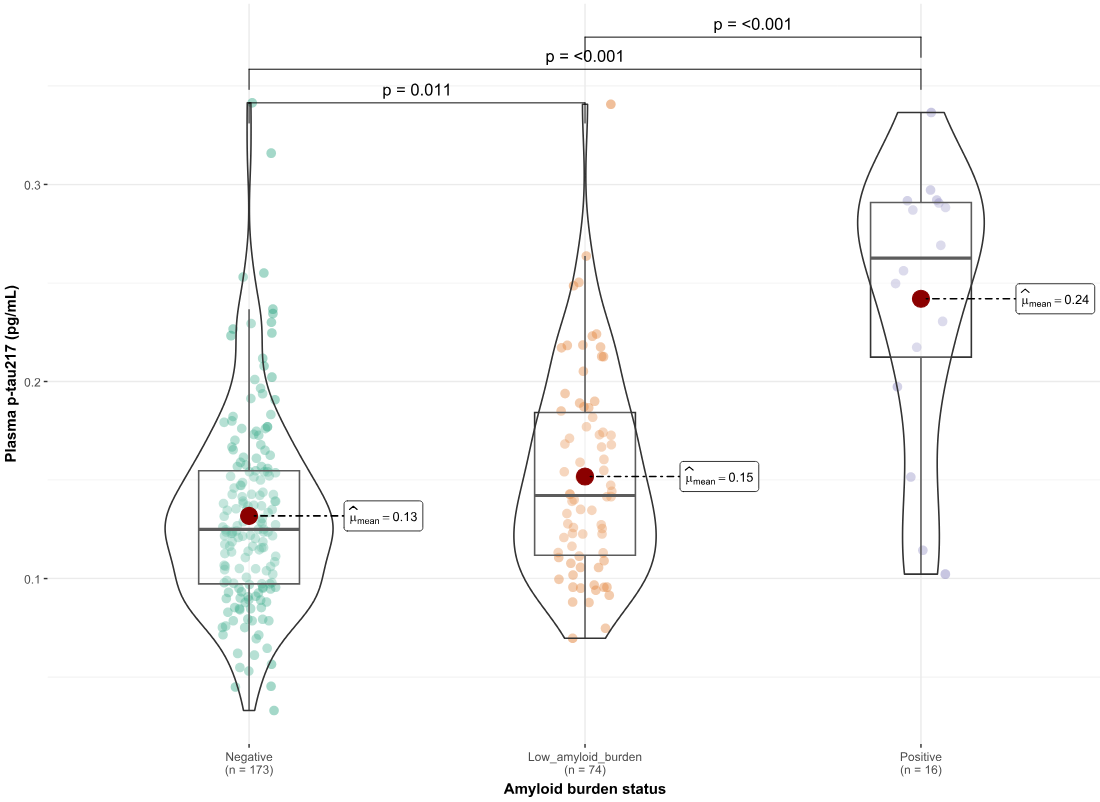


**Supplementary Fig. 12 Levels of plasma p-tau217 in cognitively unimpaired participants respectively to their amyloid burden status**

The figure shows plasma levels of the plasma biomarkers in participants classified according to their amyloid burden status (CSF A+ - Aβ42/40 < 0.071, PET A+ > 30 Centiloids). N=173 cognitively unimpaired (CU) participants were classified as negative; n=74 CU participants were classified as low amyloid burden; n=16 CU participants were classified as positive. Boxplots included in the violin plots are shown as a median and interquartile range (IQR), upper whisker is 75^th^ percentile plus 1.5 times IQR and lower whisker is 25^th^ percentile minus 1.5 IQR. Red dot represents mean of the group. Group differences were examined using Dwass-Steel-Critchlow-Fligner test.


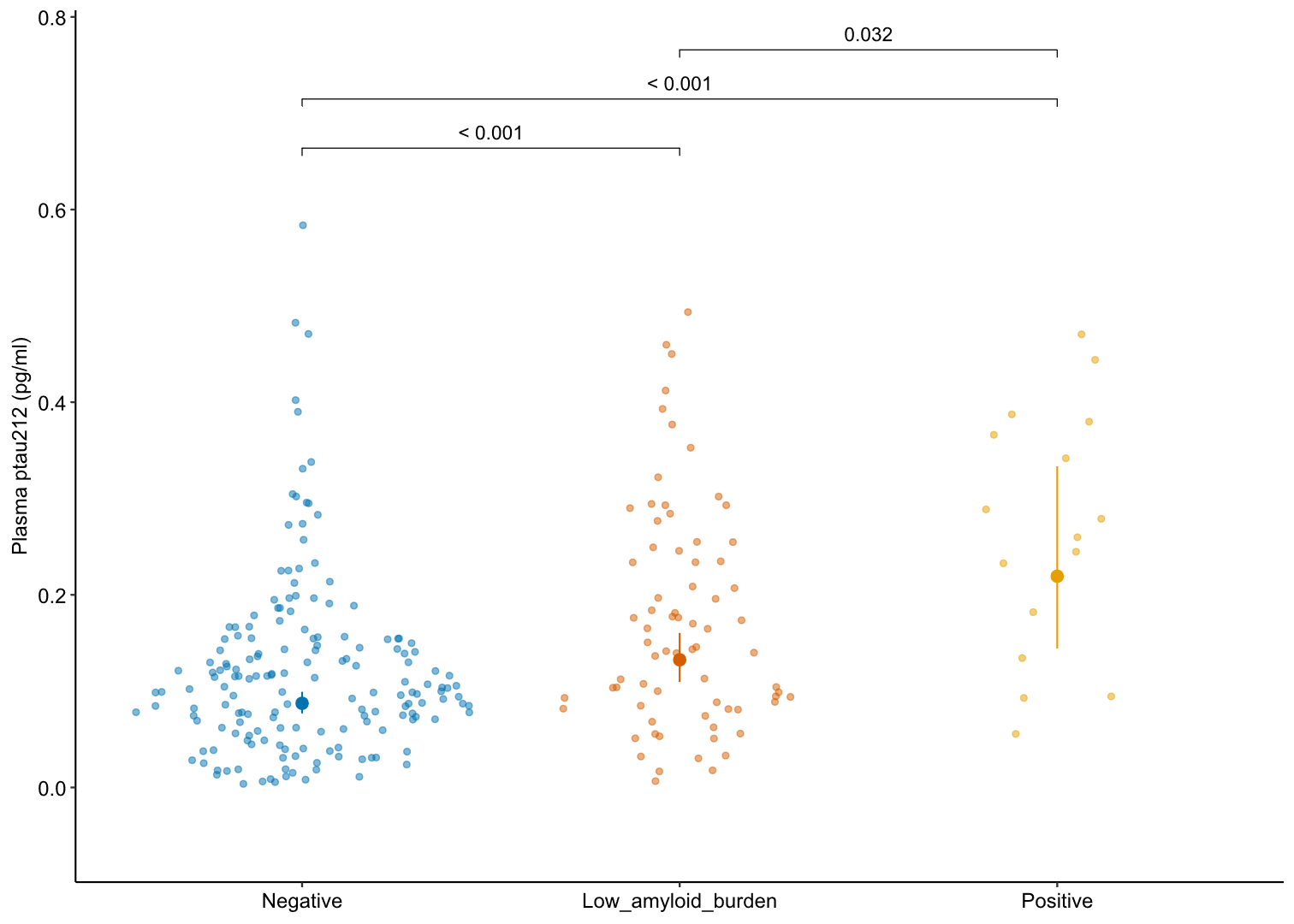


**Supplementary Fig. 13 Levels of adjusted plasma p-tau212 in cognitively unimpaired participants respectively to their amyloid burden status**

The figure shows plasma levels of the plasma biomarkers in participants classified according to their amyloid burden status (CSF A+ - Aβ42/40 < 0.071, PET A+ > 30 Centiloids). N=173 cognitively unimpaired (CU) participants were classified as negative; n=74 CU participants were classified as low amyloid burden; n=16 CU participants were classified as positive. Plots show the original data points as well as the estimated marginal means (dots) from a linear model adjusted by age, sex, Body Mass Index (BMI) and estimated Glomerular Filtration Rate (eGFR). The lines adjacent to the dots represent the 95% percent confidence interval of the estimated marginal mean, while the p values represent the pairwise contrasts of the different amyloid subgroups.


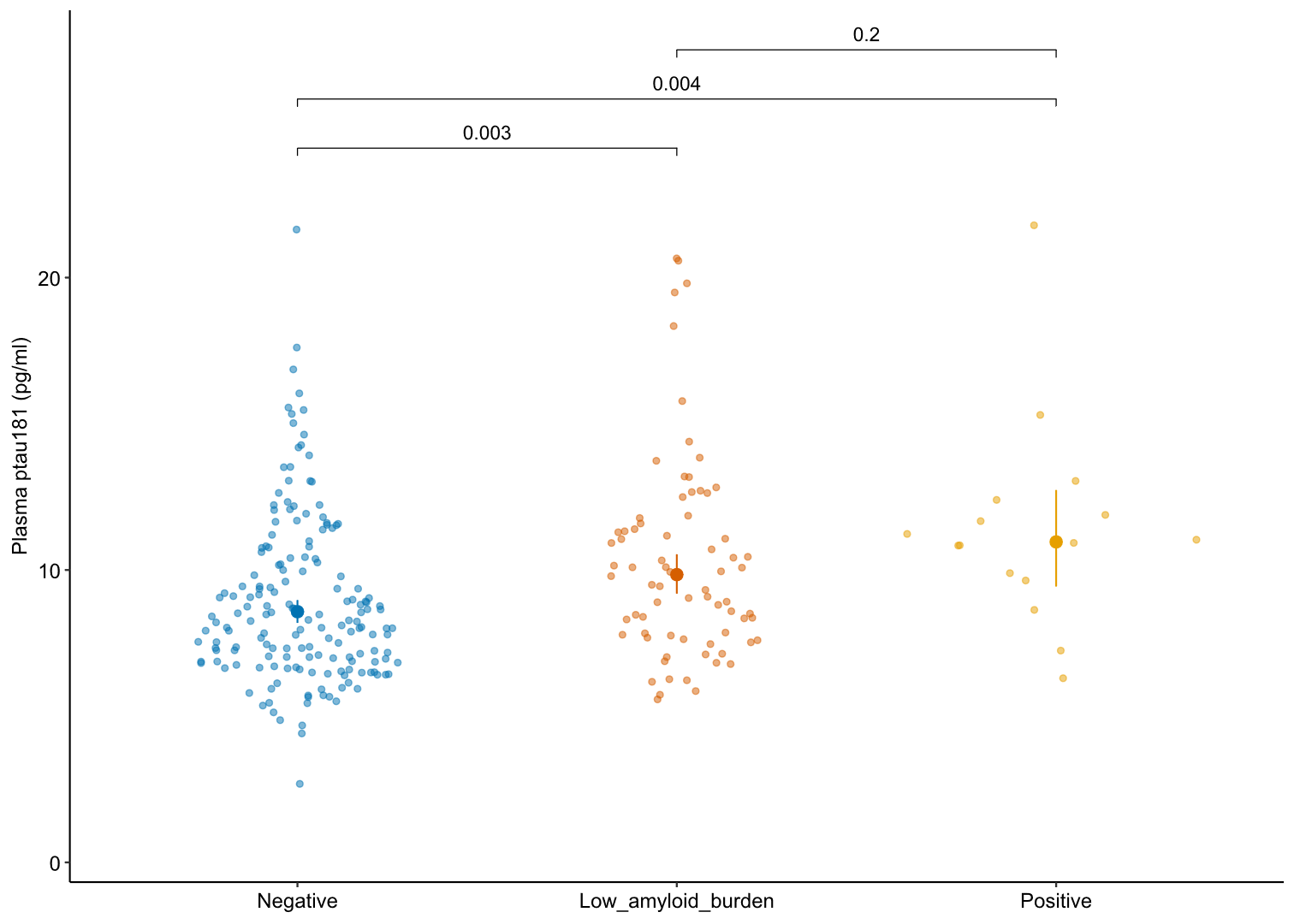


**Supplementary Fig. 14 Levels of adjusted plasma p-tau181 in cognitively unimpaired participants respectively to their amyloid burden status**

The figure shows plasma levels of the plasma biomarkers in participants classified according to their amyloid burden status (CSF A+ - Aβ42/40 < 0.071, PET A+ > 30 Centiloids). N=173 cognitively unimpaired (CU) participants were classified as negative; n=74 CU participants were classified as low amyloid burden; n=16 CU participants were classified as positive. Plots show the original data points as well as the estimated marginal means (dots) from a linear model adjusted by age, sex, Body Mass Index (BMI) and estimated Glomerular Filtration Rate (eGFR). The lines adjacent to the dots represent the 95% percent confidence interval of the estimated marginal mean, while the p values represent the pairwise contrasts of the different amyloid subgroups.


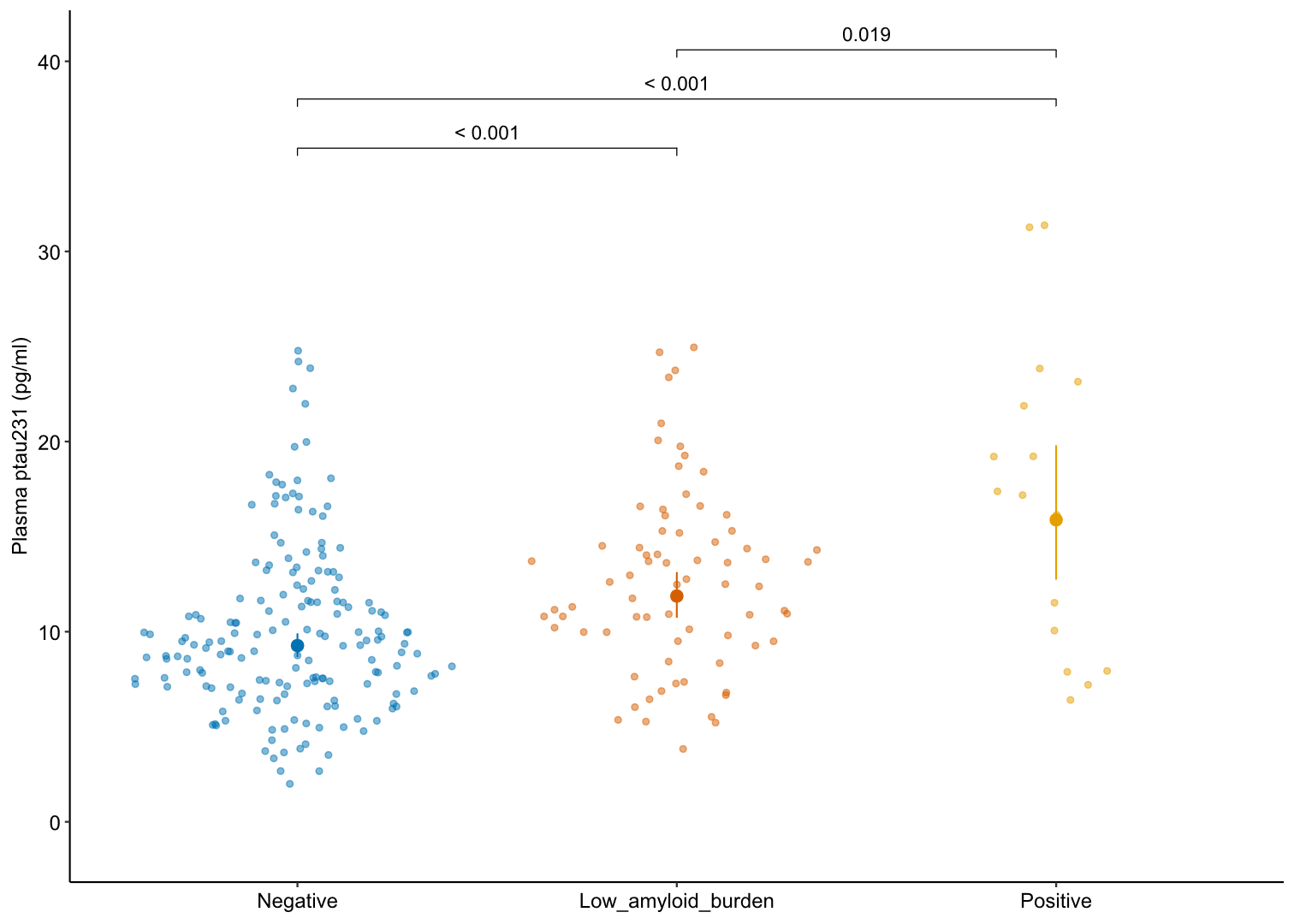


**Supplementary Fig. 15 Levels of adjusted plasma p-tau231 in cognitively unimpaired participants respectively to their amyloid burden status**

The figure shows plasma levels of the plasma biomarkers in participants classified according to their amyloid burden status (CSF A+ - Aβ42/40 < 0.071, PET A+ > 30 Centiloids). N=173 cognitively unimpaired (CU) participants were classified as negative; n=74 CU participants were classified as low amyloid burden; n=16 CU participants were classified as positive. Plots show the original data points as well as the estimated marginal means (dots) from a linear model adjusted by age, sex, Body Mass Index (BMI) and estimated Glomerular Filtration Rate (eGFR). The lines adjacent to the dots represent the 95% percent confidence interval of the estimated marginal mean, while the p values represent the pairwise contrasts of the different amyloid subgroups.


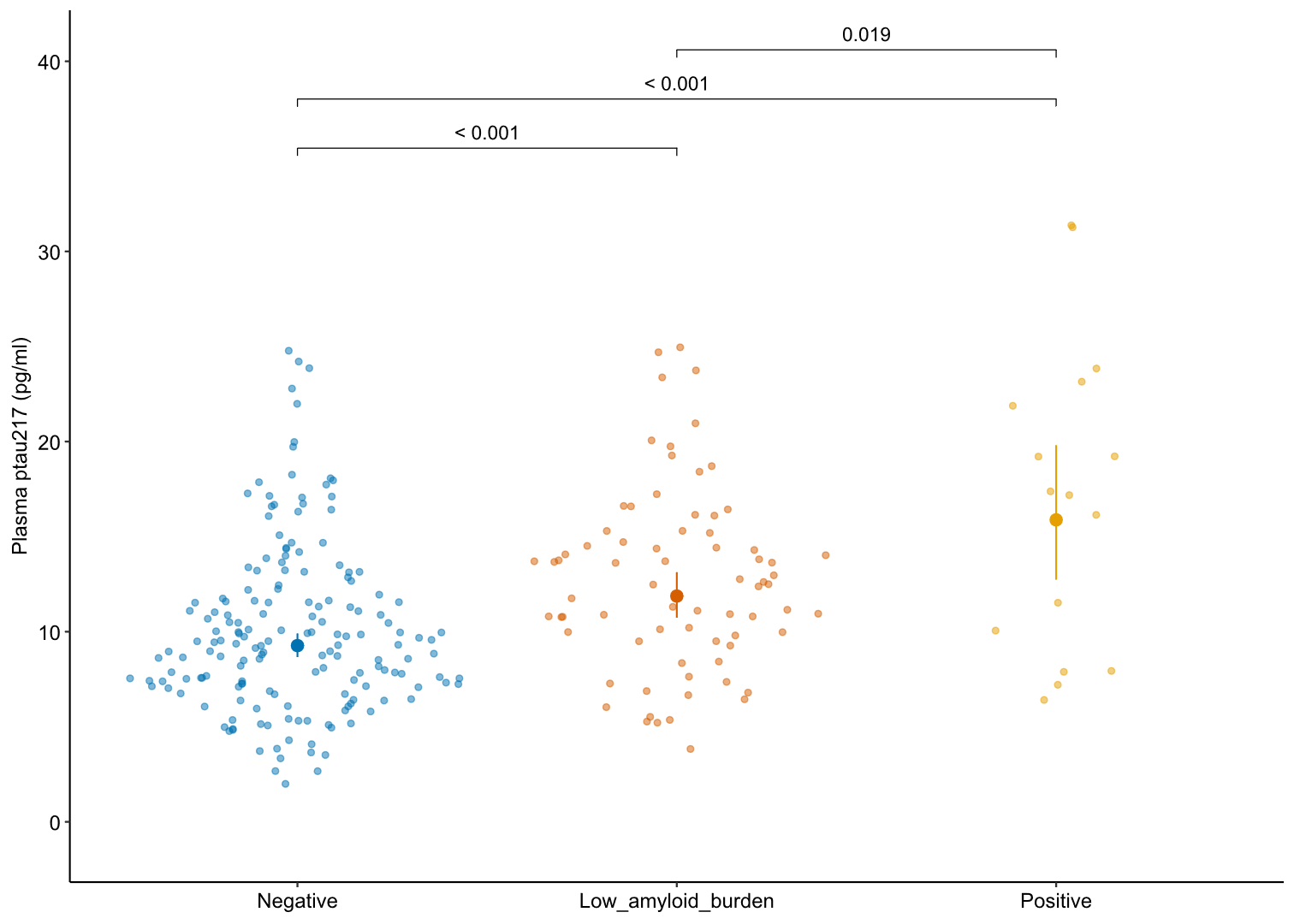


**Supplementary Fig. 16 Levels of adjusted plasma p-tau217 in cognitively unimpaired participants respectively to their amyloid burden status**

The figure shows plasma levels of the plasma biomarkers in participants classified according to their amyloid burden status (CSF A+ - Aβ42/40 < 0.071, PET A+ > 30 Centiloids). N=173 cognitively unimpaired (CU) participants were classified as negative; n=74 CU participants were classified as low amyloid burden; n=16 CU participants were classified as positive. Plots show the original data points as well as the estimated marginal means (dots) from a linear model adjusted by age, sex, Body Mass Index (BMI) and estimated Glomerular Filtration Rate (eGFR). The lines adjacent to the dots represent the 95% percent confidence interval of the estimated marginal mean, while the p values represent the pairwise contrasts of the different amyloid subgroups.


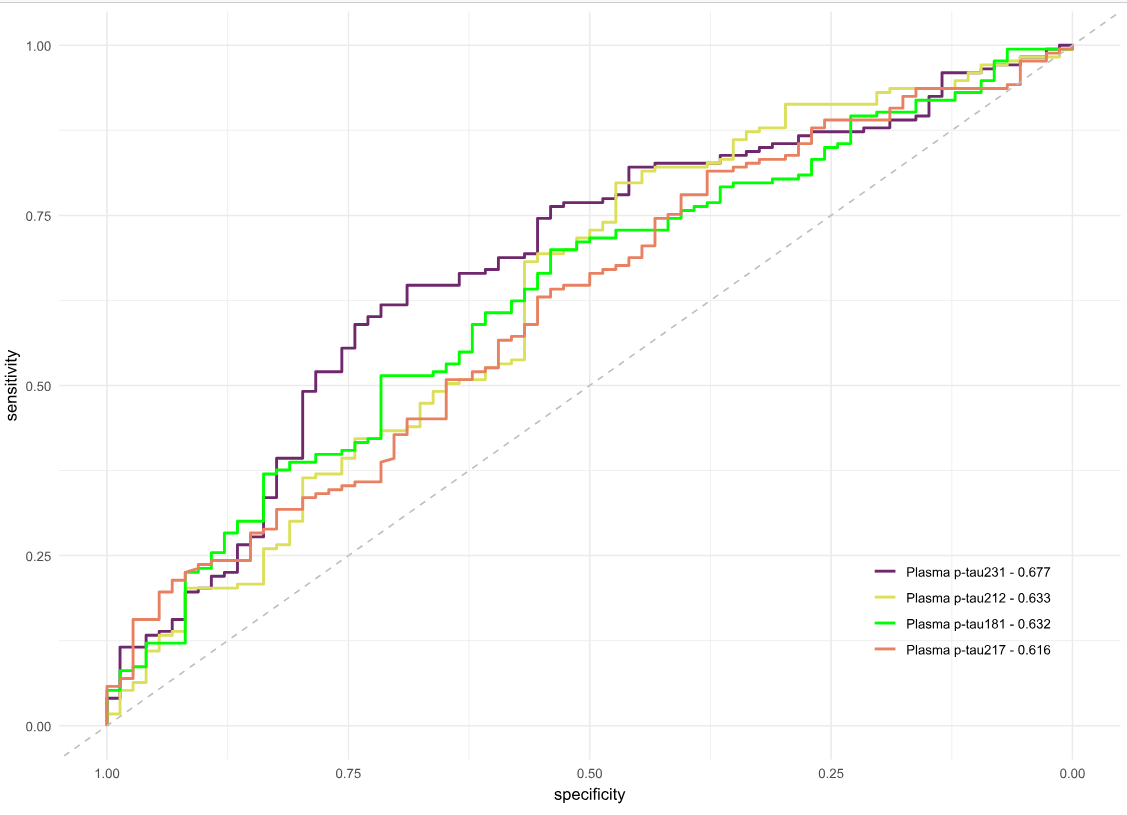


**Supplementary Fig. 17 ROC analyses of plasma biomarkers for discriminating between CSF Aβ- and low Aβ burden.**

Receiver operating characteristic curves (ROC) to discriminate between cognitively unimpaired (CU) CSF A- and low Aβ burden participants. p-tau181: AUC = 0.63 (95% CI = 0.56-0.71); p-tau212: AUC = 0.63 (95% CI = (0.55-0.71); p-tau217: AUC= 0.62 (95% CI = 0.54-0.69) and p-tau231 AUC = 0.68 (95% CI = 0.60-0.75); are on the graph.
